# Primary care referrals and abdominal imaging in people with new-onset diabetes and weight loss: cohort study using the English national sentinel network database

**DOI:** 10.1136/bmjopen-2026-119662

**Published:** 2026-08-18

**Authors:** Agnieszka Lemanska, Hugh Claridge, Claire A Price, Jessica Abell, Eithne Costello, Georgina Evans-Lilley, Stephen Pandol, Poppy Richards, Rachael Z Stolzenberg-Solomon, Simon de Lusignan

**Affiliations:** 1 Imperial Clinical Trials Unit, School of Public Health, Imperial College London, London, UK; 2 Faculty of Health and Medical Science, University of Surrey, Guildford, UK; 3 Data Science and AI Department, National Physical Laboratory, Teddington, UK; 4 National Health Service (NHS) England, National Cancer Programme, London, UK; 5 Molecular and Clinical Cancer Medicine, University of Liverpool, Liverpool, UK; 6 Department of Medicine, Cedars-Sinai Medical Center, Los Angeles, California, USA; 7 Division of Cancer Epidemiology and Genetics, National Institutes of Health, Bethesda, Maryland, USA; 8 Nuffield Department of Primary Care Health Sciences, University of Oxford, Oxford, UK

**Keywords:** Primary Health Care, Electronic Health Records, Pancreatic disease, DIABETES & ENDOCRINOLOGY, Primary Care

## Abstract

**Abstract:**

**Objectives:**

Pancreatic cancer is diagnosed in approximately 1% of individuals with new-onset diabetes, increasing to 3% among those aged ≥60 years with weight loss. UK clinical guidelines recommend abdominal imaging to rule out pancreatic cancer in this high-risk group. In June 2025, National Health Service (NHS) England launched a case-finding pilot to test approaches to improve primary care referrals for imaging in these patients and improve the quality of body weight and diabetes-related data recorded in electronic healthcare records (EHRs). This study reports referral and imaging rates among the eligible group and the availability of body weight and glycated haemoglobin (HbA1c) measurements in people with diabetes, providing baseline data prior to implementation of the NHS England pilot.

**Design:**

Retrospective cohort study.

**Setting:**

Primary care. The database of 8 million patients from 781 general practices from the Royal College of General Practitioners Research and Surveillance Centre.

**Participants:**

Adults diagnosed with diabetes between 2015 and 2021.

**Outcome measures:**

Proportions of eligible individuals who received a referral and imaging within 12 months before or after diabetes diagnosis and the number of body weight and HbA1c measurements recorded for individuals with diabetes.

**Results:**

Among 180 815 individuals diagnosed with diabetes, 92 210 (51.0%) were aged ≥60 years (median age 71, 46% women). Overall, 152 300 (84.2%) had at least one body weight and 161 631 (89.4%) had at least one HbA1c measurement recorded in EHRs. Among the 1411 patients with new-onset diabetes and weight loss, 372 (26.4%) had a referral, 141 (10.0%) imaging and 40 (2.8%) had pancreatic cancer recorded.

**Conclusions:**

This study demonstrated potential underutilisation of referral guidance and under-referral among the eligible population. The findings provide baseline data to support the launch of the NHS England pilot. Further research is needed to better understand current practice and support the implementation of cancer referral guidelines.

**STRENGTHS AND LIMITATIONS OF THIS STUDY**

- The study used a large, nationally representative primary care database, enabling a robust evaluation of clinical practice across England and providing baseline data for the NHS England pancreatic cancer case-finding pilot.
- Use of routinely collected electronic health records enabled a real-world assessment of the availability and utility of weight and glycated haemoglobin data for data-driven cancer risk tools.
- Reliance on routinely collected healthcare data may have resulted in incomplete or delayed recording of symptoms, referrals and abdominal imaging.
- The study was limited to data recorded in primary care, without linkage to secondary care or cancer registry, and this may have led to under-reporting of abdominal imaging and pancreatic cancer diagnoses.
- The retrospective observational design without information on clinicians’ decision-making limited assessment of the appropriateness of referrals and reasons for non-referral.

## Introduction

 Pancreatic cancer carries a poor prognosis, with a 5-year survival rate of less than 10%. Early diagnosis and surgical resection offer the best chance of survival, yet the absence of population-based screening limits timely detection. Targeting high-risk groups improves the balance of benefits and harms associated with screening. Diabetes plays a complex role as both a risk factor and symptom. Glucose dysregulation is commonplace in pancreatic cancer and precedes up to 80% of diagnoses,^1–3^ with pancreatic cancer inducing diabetes through disrupted insulin production.^4^ This pancreatogenic type 3c diabetes is frequently mistaken for type 2 diabetes, delaying pancreatic cancer diagnosis.^5^ Therefore, new-onset diabetes (NOD) is of particular concern, and pancreatic cancer is diagnosed in 0.5–1% of people with NOD, representing a risk approximately eight times that of the general population.^6 7^ The risk increases further to up to 3% when NOD is accompanied by unexplained weight loss, particularly in older adults.^8 9^

Diabetes is a common condition, so to make referrals for pancreatic cancer feasible and acceptable, further risk stratification is required.^9^ In the USA, the Preventive Services Task Force recognises both new-onset and pre-existing diabetes as risk factors but advises against routine screening in these groups.^10^ Unexplained weight loss, although a non-specific symptom for many cancers, becomes a clinically significant indicator for pancreatic cancer when combined with NOD.^11–13^ The US National Comprehensive Cancer Network guidelines recommend considering pancreatic cancer in patients with diabetes who present with weight loss.^14 15^ In the UK, national guidelines recommend urgent referral for abdominal imaging in individuals with weight loss and NOD if they are aged 60 and over (England and Wales)^16^ and 55 and over (Scotland).^17^ Box 1 reproduces the National Institute for Health and Care Excellence (NICE) Suspected Cancer Recognition and Referral Guideline (NG12), with NOD added in 2015 to the list of pancreatic cancer symptoms.^16^

Box 1 **Clinical guidelines by the National Institute for Health and Care Excellence^16^ indicating that people aged 60 and over with weight loss and at least one other symptom including diarrhoea, constipation, back pain, abdominal pain, nausea, vomiting and/or new-onset diabetes should be assessed for pancreatic cancer**

Consider an urgent, direct access CT scan or an urgent ultrasound scan if CT not available, to assess for pancreatic cancer in people aged 60 and over with weight loss and any of the following:

- Diarrhoea.
- Back pain.
- Abdominal pain.
- Nausea.
- Vomiting.
- Constipation.
- New-onset diabetes (added in 2015).

A randomised controlled trial is currently underway in the US testing whether referrals based on age, changes in glycaemic control and weight loss improve early detection.^18^ In 2025, the National Health Service (NHS) in England launched an early diagnosis initiative targeting individuals aged 60 and over with NOD and weight loss.^19 20^ Underpinned by the NICE NG12 guideline and implemented using a case-finding approach, the pilot enables 300 general practices to search electronic healthcare records (EHRs) to proactively identify eligible patients and refer them for imaging. NHS England is supporting the pilot with funding and resources, aiming to improve early diagnosis, survival rates and the quality of data related to weight, weight loss and diabetes.

This observational study provides supporting evidence for the implementation and evaluation of the NHS England pilot. The aim of this study was to provide baseline data to inform the NHS England pilot, for the target population in terms of (1) referral rates for suspected pancreatic cancer; (2) rates of abdominal imaging, including CT and ultrasound scans (USSs); and (3) recording of unexplained weight loss, along with the completeness of body weight and glycated haemoglobin (HbA1c) data in people with NOD.

We conducted retrospective analyses of a large, population-representative dataset. Referral rates and abdominal imaging were assessed among individuals with NOD and unexplained weight loss, along with the completeness of body weight and HbA1c data. We assessed the latter because one of the objectives of the NHS England pilot is to improve the recording of body weight and HbA1c in EHRs. Regular weight and HbA1c recordings are important because they can be used to determine eligibility for a referral. Following completion of the pilot, these analyses will be repeated to assess changes in referral and imaging rates, pancreatic cancer diagnoses and data quality, complementing the NHS England pilot evaluation with real-world evidence from practice.

## Methods

### Study design and setting

This was a retrospective cohort study in primary care. It included individuals diagnosed with diabetes between January 2015 and May 2021. The study period began in 2015 because of the implementation of the NG12 guidelines in June 2015, recognising that adoption of new guidelines is gradual rather than immediate. The end date reflected the point at which data were censored. The study provides data on referral and imaging rates, as well as the weight and HbA1c data quality, after the introduction of the NG12 guideline but prior to the start of the NHS England pilot.

### Dataset

We used a dataset from the nationally representative database of the Royal College of General Practitioners (RCGP) Research and Surveillance Centre (RSC).^4 21 22^ The dataset was extracted in May 2021 from 781 general practices, covering over 8 million patients. Only practices that met strict data quality control standards were included. Data access was provided via the Oxford–RCGP Clinical Informatics Digital Hub (ORCHID), hosted by the University of Oxford.^23^ In addition to its principal role as a sentinel surveillance network, the RSC has been actively involved in cardiometabolic research, particularly in diabetes, which is primarily managed in primary care.^24 25^ The combination of universal EHRs use, a national pay-for-performance system in place since 2004 and the ‘LabLinks’ system which automatically returns pathology results to the primary care record, makes the RCGP RSC an ideal platform for conducting this research.^26^

### Study cohort

We included all adults diagnosed with diabetes between January 2015 and May 2021. The date of diabetes diagnosis was the study index date for each participant. We stratified the cohort into two age groups, 18–59 at the index date (<60, below the NG12 referral threshold), and those aged 60 and over (≥60). We included people with both type 1 and type 2 diabetes, identified using a comprehensive ontology-based approach.^26^ Individuals with unspecified diabetes type, gestational diabetes and pancreatic cancer diagnosis before diabetes were excluded.

### Data preparation and outcome measures

We extracted information on diabetes and pancreatic cancer diagnoses for individuals in the cohort, along with demographic data including age, sex, ethnicity and deprivation. Information on cancers other than pancreatic cancer was not extracted, and individuals with other cancer diagnoses were eligible for inclusion in the study. The dates of diabetes and pancreatic cancer diagnoses were defined as the first recorded entry of the relevant clinical code. These dates were used to determine eligibility for the study cohort, and individuals with a recorded pancreatic cancer diagnosis before their diabetes diagnosis were excluded. The data curation process using the Systematised Nomenclature of Medicine clinical terms (SNOMED CT) system has been described in detail elsewhere.^26–28^ Deprivation was measured using the Index of Multiple Deprivation (IMD) from the English Indices of Deprivation 2019, a socio-economic indicator based on the patient’s or practice’s postcode. Where new postcodes were not mapped, IMD values were recorded as missing.

We extracted data on unexplained weight loss, referrals to secondary care for suspected pancreatic cancer, abdominal imaging, including CT and USSs and measurements of body weight and HbA1c within 12 months before and after the index date. A 24-month study window, comprising the 12 months before and the 12 months after the index date, was applied to account for potential delays in recording diabetes diagnoses and other clinical information in the EHRs systems. The SNOMED CT code lists were provided in the online supplemental material 1 file.

### Data analysis

We reported demographic information for the study cohort using descriptive statistics, means with 95% CIs, medians with IQRs and counts with percentages. Because ethnicity and deprivation had varying amounts of missing data, to ensure transparency, we reported the percentages of missing data for each variable separately. Percentages were calculated based on available data to provide less biased estimates of the true proportions in the population, with missing data reported on top, therefore the combined percentages for ethnicity and deprivation exceeded 100%.

To analyse the numbers of referrals and scans, we reported raw counts and calculated rates as percentages of eligible individuals. Similarly, we reported counts and percentages of individuals with at least one body weight and HbA1c measurement and those with two or more measurements. This was of interest because having two or more measurements could enable automated assessment of changes in weight and HbA1c to support the referral process.

The database was managed in Structured Query Language Server Management Studio V.18.9.1. Statistical analyses were performed using R V.4.1.0 (R Foundation for Statistical Computing, Vienna, Austria) in RStudio (Posit PBC, Boston, Massachusetts, USA). Reporting followed the Strengthening the Reporting of Observational Studies in Epidemiology (STROBE) guidelines on Strengthening the Reporting of Observational Studies.^29^

### Patient and public involvement

Patients and the public were not directly involved in the design or dissemination of this study. The RCGP RSC and ORCHID regularly consult public and patients regarding the use of their data in research. Patient and public representatives from NHS England's National Cancer Programme have supported the design and delivery of the NHS England pilot.

## Results

### Characteristics of the cohort

There were 181 055 adults diagnosed with diabetes between January 2015 and May 2021 in 781 general practices. Of these, 810 (0.4%) were diagnosed with pancreatic cancer, with 127 (0.1%) in the <60 age group and 683 (0.7%) in the ≥60 age group. The median number of new diabetes diagnoses per general practice per year was 33 (IQR 19–51), with a median of 16 (9–25) diagnoses in people <60 years old and 15 (8–25) in those ≥60. Within the 810 cancer diagnoses, 240 occurred before the diagnosis of diabetes and these individuals were excluded from further analysis.

The final study cohort consisted of 180 815 people, with 88 605 (49.0%) in the <60 age group and 92 210 (51.0%) in the ≥60 age group (table 1). The median age at diabetes diagnosis was 60 years (IQR 49– 71), 49 (42–55) in the <60 age group and 71 (65–78) in the ≥60 age group. Of the total cohort, 175 030 (96.8%) had type 2 diabetes mellitus, 101 136 (55.9%) were male and 120 919 (78.5%) of the participants were white. Following a diabetes diagnosis, 570 (0.3%) people were diagnosed with pancreatic cancer, 79 (0.1%) in the <60 age group and 491 (0.5%) in the ≥60 age group.

**Table 1:** Demographic characteristics of the study cohort; adults diagnosed with type 1 and type 2 diabetes mellitus between January 2015 and May 2021

	All participants	Age group <60	Age group ≥60
People diagnosed with diabetes, n (%)	181 055 (100.0)	88 653 (49.0)	92 402 (51.0)
Number of participants per practice per year, median (IQR)	33 (19–51)	16 (9–25)	15 (8–25)
Diagnosed with pancreatic cancer before index date and excluded, n (%)	240 (0.1)	48 (<0.1)	192 (0.2)
Study cohort, n (%) (excluding people diagnosed with pancreatic cancer before index date)	180 815 (100.0)	88 605 (49.0)	92 210 (51.0)
Age			
Mean (95% CIs)	59.8 (59.8 to 59.9)	47.2 (47.1 to 47.2)	72.0 (72.0 to 72.1)
Median (IQR)	60 (49–71)	49 (42–55)	71 (65–78)
Diabetes mellitus type, n (%)			
Type 1	5785 (3.2)	4984 (5.6)	801 (0.9)
Type 2	175 030 (96.8)	83 621 (94.4)	91 409 (99.1)
Sex, n (%)			
Female	79 679 (44.1)	37 043 (41.8)	42 636 (46.2)
Male	101 136 (55.9)	51 562 (58.2)	49 574 (53.8)
Ethnicity, n (%)			
Asian	20 731 (13.5)	15 174 (19.6)	5557 (7.2)
Black	8157 (5.3)	5913 (7.6)	2244 (2.9)
Mixed	2215 (1.4)	1602 (2.1)	613 (0.8)
Other	2091 (1.4)	1483 (1.9)	608 (0.8)
White	120 919 (78.5)	53 220 (68.8)	67 699 (88.2)
Missing	26 702 (14.8)	11 213 (12.7)	15 489 (16.8)
IMD, n (%)			
1 (most deprived)	38 784 (22.3)	23 565 (27.7)	15 128 (17.0)
2	36 045 (20.7)	19 621 (23.0)	16 424 (18.5)
3	34 840 (20.0)	16 137 (18.9)	18 703 (21.1)
4	33 212 (19.1)	13 979 (16.4)	19 233 (21.7)
5 (least deprived)	31 175 (17.9)	11 926 (14.0)	19 249 (21.7)
Missing	6759 (3.7)	3286 (3.7)	3473 (3.8)
Unexplained weight loss, n (%)	2373 (1.3)	962 (1.1)	1411 (1.5)
Diagnosed with pancreatic cancer after index date, n (%)	570 (0.3)	79 (0.1)	491 (0.5)

Missing data were present for ethnicity and IMD; these were reported alongside available data. As a result, the combined percentages may exceed 100%. Percentages were calculated based on participants with available data to represent the true proportions in the population. The index date was the date of diabetes diagnosis.

IMD, Index of Multiple Deprivation.

### Rates of referrals and scans

In the study cohort, 2373 (1.3%) individuals had unexplained weight loss recorded in their EHRs within 12 months before or after diabetes diagnosis, 962 (1.1%) in the <60 age group and 1411 (1.5%) in the ≥60 age group (table 1).

Of these 2373 individuals, 513 (21.6%) had a clinical code indicating a referral for suspected pancreatic cancer, 233 (9.8%) had a code for abdominal imaging and 43 (1.8%) had a code indicating pancreatic cancer diagnosis (table 2). Among individuals in the ≥60 age group, 372 (26.4%) had a code for referral, 141 (10.0%) had a code for abdominal imaging, including 97 (6.9%) USS and 53 (3.8%) CT, and 40 (2.8%) had pancreatic cancer recorded in their EHRs. It was possible for an individual to have a code for USS and CT scan within the study window. The number of participants with referrals exceeded the number with abdominal imaging procedures. Possible reasons for this difference include imaging performed outside the study window, referrals not resulting in imaging in secondary care or incomplete recording of imaging procedures in primary care EHRs.

**Table 2:** Numbers and percentages of participants with diabetes and unexplained weight loss who received a referral and/or underwent abdominal imaging investigation, with either USS and/or CT as recorded in their electronic healthcare records

	All participants	Age group <60	Age group ≥ 60
Weight loss, n	2373	962	1411
Referral for suspected pancreatic cancer, n (%)	513 (21.6)	141 (14.7)	372 (26.4)
Abdominal imaging, n (%)	233 (9.8)	92 (9.6)	141 (10.0)
USS, n (%)	172 (7.2)	75 (7.8)	97 (6.9)
CT scan, n (%)	77 (3.2)	24 (2.5)	53 (3.8)
Pancreatic cancer diagnosis, n (%)	43 (1.8)	3 (0.3)	40 (2.8)

The study cohort was stratified in two age groups, adults under 60 years (<60, below the NICE referral guidance age cut-off) and those aged 60 years and over (≥60, within the NICE referral criteria).

NICE, National Institute for Health and Care Excellence; USS, ultrasound scan.

### The availability of body weight and HbA1c data

Within 12 months before and after the index date, among participants of all ages, 152 300 (84.2%) had at least one body weight measurement recorded in their EHRs and 161 631 (89.4%) had at least one HbA1c measurement (table 3). When assessing the availability of two or more measurements, these figures decreased to 116 288 (64.3%) for body weight and 154 392 (85.4%) for HbA1c. Among the 92 210 participants aged 60 and over, 75 229 (81.6%) had at least one weight measurement and 81 032 (87.9%) had at least one HbA1c measurement. For two or more measurements, these percentages decreased to 57 506 (62.4%) for body weight and 77 707 (84.2%) for HbA1c.

**Table 3:** Availability of body weight and HbA1c measurements in electronic healthcare records for individuals diagnosed with diabetes, within 12 months before and after diabetes diagnosis

	All participants N=180 815	Age group <60 n=88 605	Age group ≥60 n=92 210
Body weight measurements			
At least one measurement, n (%)	152 300 (84.2)	77 071 (87.0)	75 229 (81.6)
Two or more measurements, n (%)	116 288 (64.3)	58 782 (66.3)	57 506 (62.4)
HbA1c measurements			
At least one measurement, n (%)	161 631 (89.4)	80 599 (91.0)	81 032 (87.9)
Two or more measurements, n (%)	154 392 (85.4)	76 685 (86.5)	77 707 (84.2)

HbA1c, glycated haemoglobin.

## Discussion

### Summary of principal findings

This study described how closely general practices followed the NICE NG12 pancreatic cancer referral guideline for individuals with NOD and unexplained weight loss. We found that, within 12 months before or after diabetes diagnosis, only about 26% of eligible patients were recorded as having been referred for or having received abdominal imaging, indicating potential underuse of the guideline.

This study provided baseline evidence on current healthcare practice and the accuracy of primary care data recording to support the NHS England pilot, which aims to improve adherence to referral guidelines. This study also demonstrated how routinely collected data can be used to evaluate compliance with and the impact of national guidelines and highlighted gaps in data and in current practice. This provides a foundation for NHS England and general practices to deliver interventions that support early cancer diagnosis. Future analyses will repeat this evaluation to examine changes over time and support the evaluation of the NHS England pilot.

### Strengths and weaknesses of the study

A key strength of this study was the large size and high-quality database, which provided robust primary care data for analysis and enabled a detailed evaluation of referral and imaging rates. The RCGP RSC database is generally representative^4 21 22^ of the English population, with minor over-representation in London and consequently ethnic minority groups. Our findings highlight the value of routinely collected data in supporting ongoing monitoring of guideline utilisation and underscore the need for targeted interventions to address gaps in healthcare quality. Following completion of the NHS England pilot, we plan to repeat these analyses to assess changes over time.

We acknowledge limitations inherent to coded clinical data. SNOMED CT code lists may be non-exhaustive and could miss relevant information, and referrals and abdominal imaging may not be fully documented. However, the EHRs curation methodology is continually evolving, leveraging SNOMED CT Expression Constraint Language to enhance case finding,^30^ and the observed referral and imaging rates reflect both the clinical picture and documentation practices.

We relied on data recorded in primary care, and linkage to cancer registration and secondary care datasets was not available. As a result, diagnoses of pancreatic cancer and abdominal imaging were derived from coded information in general practitioner (GP) records. This may lead to under-recording of cases and inaccuracies in dates, particularly for abdominal imaging, which may be more likely to be coded in primary care when a relevant clinical finding is identified. Similarly, recording of unintended weight loss may be subject to bias and may be more likely to be coded when there is clinical suspicion of cancer. Inclusion was restricted to practices meeting predefined data quality standards, which may have introduced selection bias if clinical practice in these practices differed systematically from those not meeting the data quality standards. Furthermore, as this was an observational study and EHRs do not capture the underlying clinical decision-making, we were unable to assess the quality and appropriateness of referrals and non-referrals (when primary care has considered a referral but ruled it out for legitimate reasons). Future evaluations should aim to examine these aspects.

### Comparison with other studies

Previous studies highlighted similar gaps, suggesting that cancer referral guidelines are not followed for a high proportion of eligible patients.^31 32^ A retrospective study by Wiering *et al* reported that 60% of patients in England presenting with common cancer red-flag symptoms were not referred in accordance with national guidelines, and nearly 4% of those non-referred were diagnosed with cancer within a year of the GP consultation.^31^ The study by Clift *et al* is the only study reporting imaging rates in the same population as this study (aged ≥60 years with NOD and weight loss).^32^ They found that, within a window of 30 days from diabetes diagnosis, only 1.3% patients received USS and 2.2% received a CT scan. In this context, our study contributes to the growing body of evidence indicating underuse of the NICE referral guidelines, highlighting gaps in healthcare delivery and under-utilisation of abdominal imaging among individuals at high risk of pancreatic cancer.

### Implications and future research

Weight loss and NOD can be early indicators of pancreatic cancer, yet changes in weight and glycaemic control are not always detected due to competing demands on both patients and clinicians.^33^ In UK primary care, body weight and HbA1c are routinely collected clinical measures. For people with diabetes, these assessments are recommended as part of annual diabetes reviews and should be recorded at least once a year, although more frequent monitoring may be undertaken according to individual clinical need. However, similar to cancer, diabetes is affected by a high level of underdiagnosis. It is estimated that over 1 million people in the UK^34 35^ and nearly 9 million in the USA^36^ have undiagnosed type 2 diabetes, representing approximately a quarter of all people with the condition. This underscores the importance of regular monitoring of body weight and HbA1c in primary care, not only to support early detection of pancreatic cancer but also to facilitate timely diagnosis of diabetes. In our study, 90% of participants had two or more HbA1c measurements within 12 months before and after diabetes diagnosis, whereas only 60% had two or more body weight recordings, despite weight measurement being a less invasive test than HbA1c. Although these proportions will be smaller in people without diagnosed diabetes, routinely collected data and digital health innovations could be used to help identify eligible patients for investigations, offering opportunities for data-driven applications in early cancer detection. Emerging approaches have combined NOD with interpretable machine learning and multiomics data to improve pancreatic cancer risk stratification, suggesting that integration of routinely collected primary care data with these methods may offer opportunities for future data-driven approaches to enhance early detection.^37^ However, their success depends on the completeness and quality of medical records. In a broader context, methodologically, this study represents an innovative approach to evaluate the impact of clinical guidelines and NHS interventions, an approach which is in line with the digitisation targets of the NHS 10-year plan (2025).

Further research is needed to explore the clinical decision-making underlying non-referrals, as well as to identify barriers and facilitators to guideline implementation to ensure timely referrals for eligible patients. Individuals with NOD represent a high-risk group for pancreatic cancer, making it important to distinguish between new-onset and long-standing diabetes. While long-standing diabetes confers a modest risk increase of around 1.5-fold, NOD is associated with a sixfold to eightfold higher risk compared with the general population. Monitoring weight loss in newly diagnosed individuals could therefore refine the stratification of high-risk patients who may qualify for diagnostic imaging. A recent study by Chari *et al* confirmed the increased 3-year incidence of pancreatic cancer following NOD and identified important racial and ethnic differences in risk.^38^ These findings support the use of primary care data for further risk stratification based on demographic characteristics and targeted surveillance of groups at high risk. Future analyses should examine variation in referral and abdominal imaging rates by sex, ethnicity, geographical region and IMD to identify demographic factors associated with guideline adherence and opportunities to improve equitable implementation. Importantly, clinicians should not delay referral by waiting for a confirmed diabetes diagnosis in patients presenting with unexplained weight loss, and the NHS England pilot goes beyond current NICE guidelines and includes patients with just one elevated HbA1c without a formal NOD diagnosis coded in EHRs. Although a single elevated HbA1c result does not constitute a formal diagnosis of diabetes,^39^ it remains a critical clinical indicator that should, in practice, prompt adherence to clinical guidelines supporting further investigation.

## Conclusions

This study demonstrated opportunities to improve referral rates for people with NOD and weight loss. It suggested potential underutilisation of NICE referral guidelines and a possible gap in care and data quality. In June 2025, NHS England launched an initiative to test ways to improve primary care referrals for abdominal imaging to assess for pancreatic cancer in high-risk patients. We will repeat these analyses as the NHS England pilot progresses to evaluate improvements in healthcare. Further research is needed to evaluate and improve NICE guideline implementation, ensuring more patients benefit from timely, high-quality primary care referrals.

## Supplementary material

10.1136/bmjopen-2026-119662 online supplemental file 1

## Data Availability

Data may be obtained from a third party and are not publicly available.
