# Supplementary material for: Primary care referrals and abdominal imaging in people with new-onset diabetes and weight loss: cohort study using the English national sentinel network database
Source: BMJ Open. 2026 Aug 18;16(8):e119662. doi: 10.1136/bmjopen-2026-119662 (PMC13504520; doi:10.1136/bmjopen-2026-119662)
Supplement: Supplementary file 1 [file bmjopen-16-8-s001.pdf]

Supplementary Matrial 1: SNOMED CT codelists

Pancreatic cancer

cancer_groups_pancreas	254613007	Carcinoid tumor of pancreas (disorder)
cancer_groups_pancreas	254611009	Malignant tumor of endocrine pancreas (disorder)
cancer_groups_pancreas	254609000	Carcinoma of ampulla of Vater (disorder)
cancer_groups_pancreas	253005002	Vasoactive intestinal peptide-secreting tumor (disorder)
cancer_groups_pancreas	254612002	Carcinoma of endocrine pancreas (disorder)
cancer_groups_pancreas	302822000	Insulinoma (disorder)
cancer_groups_pancreas	1259682002	Primary infiltrating duct carcinoma of pancreas (disorder)
cancer_groups_pancreas	302823005	Glucagonoma (disorder)
cancer_groups_pancreas	1651000000000	Primary adenocarcinoma of pancreas (disorder)
cancer_groups_pancreas	1259800005	Primary malignant gastrinoma of pancreas (disorder)
cancer_groups_pancreas	1259799006	Primary malignant glucagonoma of pancreas (disorder)
cancer_groups_pancreas	1259747009	Primary malignant insulinoma (disorder)
cancer_groups_pancreas	1259700000	Primary mucinous cystic neoplasm with invasive carcinoma of pancreas (disorder)
cancer_groups_pancreas	1259539007	Primary carcinoma of pancreas (disorder)
cancer_groups_pancreas	1259415000	Primary carcinoma of head of pancreas (disorder)
cancer_groups_pancreas	1259310006	Primary carcinoma of tail of pancreas (disorder)
cancer_groups_pancreas	1259311005	Primary cystadenocarcinoma of pancreas (disorder)
cancer_groups_pancreas	1259358002	Primary carcinoma of endocrine pancreas (disorder)
cancer_groups_pancreas	783771003	Acinar cell carcinoma of pancreas (disorder)
cancer_groups_pancreas	1259309001	Primary carcinoma of body of pancreas (disorder)
cancer_groups_pancreas	1187519007	Malignant gastrinoma of pancreas (disorder)
cancer_groups_pancreas	1187521002	Malignant glucagonoma of pancreas (disorder)
cancer_groups_pancreas	255039001	Pancreatic polypeptidoma (disorder)
cancer_groups_pancreas	1186869002	Neuroendocrine carcinoma of pancreas (disorder)
cancer_groups_pancreas	1186735007	Mixed neuroendocrine-non neuroendocrine neoplasm of pancreas (disorder)
cancer_groups_pancreas	770602005	Squamous cell carcinoma of exocrine pancreas (disorder)
cancer_groups_pancreas	879991002	Gastrinoma of pancreas (disorder)
cancer_groups_pancreas	783706007	Serous cystadenocarcinoma of pancreas (disorder)
cancer_groups_pancreas	1197279000	Primary malignant solid pseudopapillary neoplasm of pancreas (disorder)
cancer_groups_pancreas	1237341006	Intraductal tubulopapillary malignant neoplasm of pancreas (disorder)
cancer_groups_pancreas	715414009	Familial malignant neoplasm of pancreas (disorder)
cancer_groups_pancreas	866048009	Mucinous cystic neoplasm with invasive carcinoma of pancreas (disorder)
cancer_groups_pancreas	733351008	Primary malignant neuroendocrine neoplasm of ampulla of Vater (disorder)
cancer_groups_pancreas	681721000000000	Primary adenocarcinoma of head of pancreas (disorder)
cancer_groups_pancreas	681621000000000	Primary adenocarcinoma of body of pancreas (disorder)
cancer_groups_pancreas	717919005	Neuroendocrine tumor of pancreas (disorder)
cancer_groups_pancreas	782697005	Solid pseudopapillary carcinoma of pancreas (disorder)
cancer_groups_pancreas	1141626005	Adenocarcinoma of pancreas with neuregulin 1 gene fusion (disorder)
cancer_groups_pancreas	1197283000	Primary mixed acinar endocrine-ductal carcinoma of pancreas (disorder)
cancer_groups_pancreas	1208744003	Non-functioning neuroendocrine neoplasm of pancreas (disorder)
cancer_groups_pancreas	1208745002	Serotonin-producing neuroendocrine neoplasm of pancreas (disorder)
cancer_groups_pancreas	93715005	Primary malignant neoplasm of body of pancreas (disorder)
cancer_groups_pancreas	109848009	Overlapping malignant neoplasm of pancreas (disorder)
cancer_groups_pancreas	1197286008	Primary mixed acinar ductal carcinoma of pancreas (disorder)
cancer_groups_pancreas	1565075100011910	Primary sarcoma of pancreas (disorder)
cancer_groups_pancreas	691151000000000	Ca pancreas NOS (disorder)
cancer_groups_pancreas	682821000000000	Benign neuroendocrine neoplasm of pancreas (disorder)
cancer_groups_pancreas	866052009	Mixed ductal-neuroendocrine carcinoma of pancreas (disorder)
cancer_groups_pancreas	556041000000000	Malignant neoplasm of pancreas NOS (disorder)
cancer_groups_pancreas	556031000000000	Malignant neoplasm of specified site of pancreas NOS (disorder)
cancer_groups_pancreas	1279841001	Undifferentiated carcinoma with osteoclast-like giant cells of pancreas (disorder)
cancer_groups_pancreas	1269437009	Glucagonoma of uncertain behavior (disorder)
cancer_groups_pancreas	1268698000	Primary solid pseudopapillary carcinoma of pancreas (disorder)
cancer_groups_pancreas	1268563005	Primary acinar cell carcinoma of pancreas (disorder)
cancer_groups_pancreas	1268561007	Primary serous cystadenocarcinoma of pancreas (disorder)
cancer_groups_pancreas	1268546006	Primary mucinous cystadenocarcinoma of pancreas (disorder)
cancer_groups_pancreas	1268542008	Primary invasive intraductal papillary-mucinous carcinoma of pancreas (disorder)
cancer_groups_pancreas	255088001	Malignant tumor of exocrine pancreas (disorder)
cancer_groups_pancreas	1290117000	Parathyroid hormone-related peptide-secreting tumor of pancreas (disorder)
cancer_groups_pancreas	1290116009	Gastric inhibitory peptide-secreting tumor of pancreas (disorder)
cancer_groups_pancreas	1290066001	Well-differentiated neuroendocrine tumor of ampulla of Vater (disorder)
cancer_groups_pancreas	1287665004	Primary well-differentiated neuroendocrine tumor of pancreas (disorder)
cancer_groups_pancreas	1287657002	Primary well-differentiated neuroendocrine tumor of ampulla of Vater (disorder)
cancer_groups_pancreas	1682394100011910	Primary malignant gastrointestinal stromal neoplasm of pancreas (disorder)
cancer_groups_pancreas	682611000000000	Benign carcinoid tumor of pancreas (disorder)
cancer_groups_pancreas	189814006	Pancreatoblastoma (disorder)
cancer_groups_pancreas	352701000000000	Primary malignant neoplasm of neck of pancreas (disorder)
cancer_groups_pancreas	681911000000000	Primary adenocarcinoma of pancreatic duct (disorder)
cancer_groups_pancreas	681831000000000	Primary adenocarcinoma of neck of pancreas (disorder)

cancer_groups_pancreas	681971000000000	Primary adenocarcinoma of tail of pancreas (disorder)
cancer_groups_pancreas	721718003	Primary adenocarcinoma of ampulla of Vater (disorder)
cancer_groups_pancreas	785879009	Mucinous cystadenocarcinoma of pancreas (disorder)
cancer_groups_pancreas	94082003	Primary malignant neoplasm of tail of pancreas (disorder)
cancer_groups_pancreas	700423003	Adenocarcinoma of pancreas (disorder)
cancer_groups_pancreas	93939009	Primary malignant neoplasm of pancreatic duct (disorder)
cancer_groups_pancreas	326072005	Carcinoma of head of pancreas (disorder)
cancer_groups_pancreas	735735001	Primary malignant neuroendocrine neoplasm of pancreas (disorder)
cancer_groups_pancreas	314947008	Carcinoid tumor of ampulla of Vater (disorder)
cancer_groups_pancreas	363417006	Malignant tumor of ampulla of Vater (disorder)
cancer_groups_pancreas	371967001	Primary malignant neoplasm of ampulla of Vater (disorder)
cancer_groups_pancreas	363418001	Malignant tumor of pancreas (disorder)
cancer_groups_pancreas	363419009	Malignant tumor of head of pancreas (disorder)
cancer_groups_pancreas	235965006	Malignant cystic tumor of exocrine pancreas (disorder)
cancer_groups_pancreas	235966007	Cystadenocarcinoma of pancreas (disorder)
cancer_groups_pancreas	363368005	Carcinoma of body of pancreas (disorder)
cancer_groups_pancreas	363369002	Carcinoma of tail of pancreas (disorder)
cancer_groups_pancreas	255038009	Growth hormone releasing factor-secreting tumor (disorder)
cancer_groups_pancreas	1268911008	Primary pancreatoblastoma (disorder)
cancer_groups_pancreas	255040004	Parathyroid hormone-related peptide-secreting tumor (disorder)
cancer_groups_pancreas	255041000	Human pancreatic polypeptide-secreting tumor (disorder)
cancer_groups_pancreas	1268532006	Primary carcinoma of ampulla of Vater (disorder)
cancer_groups_pancreas	314964006	Local recurrence of malignant tumor of pancreas (disorder)
cancer_groups_pancreas	255042007	Gastric inhibitory peptide-secreting tumor (disorder)
cancer_groups_pancreas	372003004	Primary malignant neoplasm of pancreas (disorder)
cancer_groups_pancreas	93843007	Primary malignant neoplasm of islets of Langerhans (disorder)
cancer_groups_pancreas	372119009	Primary malignant neoplasm of head of pancreas (disorder)
cancer_groups_pancreas	372142002	Carcinoma of pancreas (disorder)
cancer_groups_pancreas	780821007	Invasive intraductal papillary-mucinous carcinoma of pancreas (disorder)
cancer_groups_pancreas	1148551007	Infiltrating duct carcinoma of pancreas (disorder)
cancer_groups_pancreas	546831000000000	Malignant neoplasm of other specified sites of pancreas (disorder)
cancer_groups_pancreas	713189001	Malignant insulinoma (disorder)
cancer_groups_pancreas	792907004	Adenocarcinoma of pancreatic duct (disorder)
cancer_groups_pancreas	269556009	Ca pancreas NOS (disorder)
cancer_groups_pancreas	187794005	Malignant tumor of Islets of Langerhans (disorder)
cancer_groups_pancreas	187798008	Malignant neoplasm of ectopic pancreatic tissue (disorder)
cancer_groups_pancreas	187800001	Malignant neoplasm of pancreas NOS (disorder)
cancer_groups_pancreas	187799000	Malignant neoplasm of specified site of pancreas NOS (disorder)
cancer_groups_pancreas	187796007	Malignant neoplasm of other specified sites of pancreas (disorder)
cancer_groups_pancreas	187793004	Malignant tumor of pancreatic duct (disorder)
cancer_groups_pancreas	187792009	Malignant tumor of tail of pancreas (disorder)
cancer_groups_pancreas	187791002	Malignant tumor of body of pancreas (disorder)
cancer_groups_pancreas	187786003	Malignant neoplasm of sphincter of Oddi (disorder)
cancer_groups_pancreas	788390005	Benign insulinoma (disorder)
cancer_groups_pancreas	1290080008	Neuroendocrine neoplasm of pancreas (disorder)
cancer_groups_pancreas	1290115008	Growth hormone releasing factor-secreting tumor of pancreas (disorder)

Abdominal Scans: CT

cancer_tests_CT_abdo	182881000000000	CT angiography of abdomen (procedure)
cancer_tests_CT_abdo	193631000000000	CT and biopsy of upper abdomen (procedure)
cancer_tests_CT_abdo	193611000000000	CT of upper abdomen (procedure)
cancer_tests_CT_abdo	709665009	Computed tomography of stomach (procedure)
cancer_tests_CT_abdo	193531000000000	CT and drainage of pancreas (procedure)
cancer_tests_CT_abdo	182891000000000	CT angiography of abdominal aorta (procedure)
cancer_tests_CT_abdo	183051000000000	CT and drainage of abdomen (procedure)
cancer_tests_CT_abdo	183921000000000	CT of biliary tract (procedure)
cancer_tests_CT_abdo	709697009	Computed tomography of ileal conduit (procedure)
cancer_tests_CT_abdo	73487002	Unilateral tomographic imaging of kidney (situation)
cancer_tests_CT_abdo	193581000000000	CT and biopsy of liver (procedure)
cancer_tests_CT_abdo	183971000000000	CT of chest and abdomen (procedure)
cancer_tests_CT_abdo	193351000000000	CT cholangiogram (procedure)
cancer_tests_CT_abdo	193641000000000	CT and drainage of upper abdomen (procedure)
cancer_tests_CT_abdo	711278009	Computed tomography of head, neck, thorax and abdomen (procedure)
cancer_tests_CT_abdo	711309003	Computed tomography of sinus of abdomen (procedure)
cancer_tests_CT_abdo	711310008	Computed tomography of sinus of abdomen with contrast (procedure)
cancer_tests_CT_abdo	1187246003	Computed tomography of abdominal aorta without contrast (procedure)
cancer_tests_CT_abdo	184911000000000	Virtual CT colonoscopy (procedure)
cancer_tests_CT_abdo	193601000000000	CT and drainage of liver (procedure)
cancer_tests_CT_abdo	303670004	Computed tomography of abdominal organs (procedure)
cancer_tests_CT_abdo	396101000000000	Computed tomography of abdomen without contrast (procedure)
cancer_tests_CT_abdo	16440141000119108	Computed tomography of bilateral kidneys without contrast (procedure)
cancer_tests_CT_abdo	426101000000000	Computed tomography of adrenal gland without contrast (procedure)
cancer_tests_CT_abdo	869111000000000	Insertion of drain into abdomen using cone beam computed tomography guidance (procedure)
cancer_tests_CT_abdo	868931000000000	Biopsy of abdomen using cone beam computed tomography guidance (procedure)
cancer_tests_CT_abdo	868801000000000	Cone beam computed tomography of abdomen (procedure)
cancer_tests_CT_abdo	303671000	Computed tomography of abdominal vascular structure with contrast (procedure)
cancer_tests_CT_abdo	241554003	Computed tomography arterioportography with contrast (procedure)
cancer_tests_CT_abdo	859191000000000	Single photon emission computed tomography with computed tomography of liver using technetium 99m macroaggregated albumin (procedure)
cancer_tests_CT_abdo	432905004	Computed tomography of liver and portal vein with contrast (procedure)
cancer_tests_CT_abdo	16326461000119108	Computed tomography angiography of chest and abdomen with contrast (procedure)
cancer_tests_CT_abdo	16321631000119108	Computed tomography angiography of neck and chest and abdomen with contrast (procedure)
cancer_tests_CT_abdo	925101000000000	Single photon emission computed tomography using iodine 123 meta-iodobenzylguanidine with computed tomography of abdomen (procedure)
cancer_tests_CT_abdo	927171000000000	Single photon emission computed tomography using indium 111 labelled octreotide with computed tomography of abdomen (procedure)
cancer_tests_CT_abdo	16534101000119106	Single photon emission computed tomography with computed tomography of liver using technetium Tc^99m^ sulfur colloid (procedure)
cancer_tests_CT_abdo	927271000000000	Single photon emission computed tomography using gallium 67 with computed tomography of abdomen (procedure)
cancer_tests_CT_abdo	1196926008	Computed tomography triple phase study of pancreas (procedure)
cancer_tests_CT_abdo	1196927004	Computed tomography triple phase study of abdomen with contrast (procedure)
cancer_tests_CT_abdo	1196929001	Computed tomography dual phase study of abdomen with contrast (procedure)
cancer_tests_CT_abdo	1196930006	Computed tomography dual phase study of kidney (procedure)
cancer_tests_CT_abdo	1065551000000101	Computed tomography nephrostography (procedure)
cancer_tests_CT_abdo	717691006	Insertion of drain into abdomen using cone beam computed tomography guidance (procedure)
cancer_tests_CT_abdo	1077001000000109	Computed tomography of stomach with contrast (procedure)
cancer_tests_CT_abdo	419812000	Computed tomography and drainage of upper abdomen (procedure)
cancer_tests_CT_abdo	752511000000000	Computed tomography triple phase study of kidney (procedure)
cancer_tests_CT_abdo	420199004	Computed tomography and biopsy of upper abdomen (procedure)
cancer_tests_CT_abdo	1084881000000108	Percutaneous irreversible electroporation ablation of neoplasm of pancreas using computed tomography guidance (procedure)
cancer_tests_CT_abdo	427101000000000	Computed tomography of chest and abdomen without contrast (procedure)
cancer_tests_CT_abdo	448677000	Computed tomography perfusion study of liver (procedure)
cancer_tests_CT_abdo	420102002	Computed tomography of upper abdomen (procedure)
cancer_tests_CT_abdo	310121000000000	Computerised tomography of adrenal gland with contrast (procedure)
cancer_tests_CT_abdo	448676009	Computed tomography perfusion study of abdomen (procedure)
cancer_tests_CT_abdo	418158005	Computed tomography and drainage of liver (procedure)
cancer_tests_CT_abdo	448760006	Computed tomography of head and neck and thorax and abdomen with contrast (procedure)
cancer_tests_CT_abdo	1208476009	Computed tomography quadruple phase study of abdomen with contrast (procedure)
cancer_tests_CT_abdo	418749009	Computed tomography and biopsy of liver (procedure)
cancer_tests_CT_abdo	419178009	Computed tomography and drainage of pancreas (procedure)
cancer_tests_CT_abdo	316041000000000	Computerised tomography of neck, thorax and abdomen with contrast (procedure)
cancer_tests_CT_abdo	419995000	Computed tomography cholangiogram (procedure)
cancer_tests_CT_abdo	316101000000000	Computerised tomography of pancreas with contrast (procedure)
cancer_tests_CT_abdo	419729006	Computed tomography angiography of abdomen (procedure)
cancer_tests_CT_abdo	418199004	Computed tomography and drainage of abdomen (procedure)
cancer_tests_CT_abdo	418863006	Computed tomography of biliary tract (procedure)
cancer_tests_CT_abdo	316231000000000	Computerised tomography of thorax and abdomen with contrast (procedure)
cancer_tests_CT_abdo	315991000000000	Computerised tomography of liver with contrast (procedure)
cancer_tests_CT_abdo	418891003	Computed tomography of chest and abdomen (procedure)
cancer_tests_CT_abdo	316061000000000	Computerised tomography of neck, thorax and abdomen (procedure)
cancer_tests_CT_abdo	717995000	Biopsy of abdomen using cone beam computed tomography guidance (procedure)
cancer_tests_CT_abdo	718013006	Cone beam computed tomography of abdomen (procedure)
cancer_tests_CT_abdo	315981000000000	Computerised tomography of kidney with contrast (procedure)
cancer_tests_CT_abdo	433270008	Computed tomography of neck, thorax and abdomen with contrast (procedure)
cancer_tests_CT_abdo	429862006	Computed tomography of liver with contrast (procedure)
cancer_tests_CT_abdo	429873004	Computed tomography of pancreas with contrast (procedure)
cancer_tests_CT_abdo	523351000000000	Computed tomography of head, thorax and abdomen (procedure)
cancer_tests_CT_abdo	429864007	Computed tomography of thorax and abdomen with contrast (procedure)
cancer_tests_CT_abdo	430439002	Computed tomography of neck, thorax and abdomen (procedure)
cancer_tests_CT_abdo	523171000000000	Computed tomography of head, thorax and abdomen with contrast (procedure)
cancer_tests_CT_abdo	322301000000000	Computerised tomography of liver and portal vein (procedure)
cancer_tests_CT_abdo	241549007	Computed tomography of liver (procedure)
cancer_tests_CT_abdo	241550007	Computed tomography of adrenals (procedure)
cancer_tests_CT_abdo	868212005	Computed tomography of chest and upper abdomen (procedure)
cancer_tests_CT_abdo	868213000	Computed tomography of chest and upper abdomen with contrast (procedure)
cancer_tests_CT_abdo	241551006	Computed tomography of pancreas (procedure)
cancer_tests_CT_abdo	241552004	Computed tomography of spleen (procedure)
cancer_tests_CT_abdo	418714002	Virtual computed tomography colonoscopy (procedure)
cancer_tests_CT_abdo	429931008	Computed tomography of kidney with contrast (procedure)
cancer_tests_CT_abdo	1111801000000107	Computed tomography triple phase of bilateral kidneys with contrast (procedure)
cancer_tests_CT_abdo	241553009	Computed tomography of abdominal aorta (procedure)
cancer_tests_CT_abdo	1110561000000100	Computed tomography portal venography of thorax and abdomen with contrast (procedure)
cancer_tests_CT_abdo	1095291000000109	Computed tomography triple phase study of pancreas (procedure)
cancer_tests_CT_abdo	1111441000000108	Computed tomography triple phase of bilateral adrenal glands with contrast (procedure)
cancer_tests_CT_abdo	1111001000000103	Computed tomography dual phase of bilateral kidneys with contrast (procedure)
cancer_tests_CT_abdo	1110571000000107	Computed tomography four phase scan of liver with contrast (procedure)
cancer_tests_CT_abdo	1108751000000107	Computed tomography of bilateral adrenal glands with contrast washout (procedure)
cancer_tests_CT_abdo	1099161000000107	Single photon emission computed tomography with computed tomography hepatobiliary study (procedure)
cancer_tests_CT_abdo	169070004	Computed tomography of abdomen (procedure)
cancer_tests_CT_abdo	440514007	Computed tomography dual phase study of pancreas (procedure)
cancer_tests_CT_abdo	438591004	Computed tomography triple phase study of liver (procedure)
cancer_tests_CT_abdo	438305004	Computed tomography dual phase study of liver (procedure)
cancer_tests_CT_abdo	1343540007	Computed tomography of gallbladder (procedure)
cancer_tests_CT_abdo	1343539005	Computed tomography of gallbladder with contrast (procedure)
cancer_tests_CT_abdo	407971000000000	Percutaneous needle biopsy of liver using computed tomography guidance (procedure)
cancer_tests_CT_abdo	16534151000119105	Single photon emission computed tomography with computed tomography of liver (procedure)
cancer_tests_CT_abdo	408031000000000	Percutaneous needle biopsy of pancreas using computed tomography guidance (procedure)
cancer_tests_CT_abdo	1337004002	Computed tomography of spleen with contrast (procedure)
cancer_tests_CT_abdo	713561006	Single photon emission computed tomography with computed tomography of abdomen using indium (111-In) radiolabeled octreotide (procedure)
cancer_tests_CT_abdo	765476000	Computed tomography of left kidney (procedure)
cancer_tests_CT_abdo	765477009	Computed tomography of right kidney (procedure)

cancer_tests_CT_abdo	281861000000000	Computed tomography of colon (procedure)
cancer_tests_CT_abdo	282821000000000	Computed tomography of abdomen NEC (procedure)
cancer_tests_CT_abdo	425666002	Computed tomography of colon (procedure)
cancer_tests_CT_abdo	444633001	Computed tomography of head, thorax and abdomen (procedure)
cancer_tests_CT_abdo	444758004	Computed tomography of head, thorax and abdomen with contrast (procedure)
cancer_tests_CT_abdo	445712007	Computed tomography of abdomen with ethiodized oil contrast (procedure)
cancer_tests_CT_abdo	446224008	Computed tomography triple phase study of kidney (procedure)
cancer_tests_CT_abdo	291981000000000	Computed tomography of abdomen NEC
cancer_tests_CT_abdo	292081000000000	Computed tomography of colon
cancer_tests_CT_abdo	292291000000000	Computed tomography of kidneys
cancer_tests_CT_abdo	345311000000000	Drainage of abdomen using computed tomography guidance (procedure)
cancer_tests_CT_abdo	345411000000000	Biopsy of liver using computed tomography guidance (procedure)
cancer_tests_CT_abdo	356491000000000	Computed tomography dual phase study of pancreas (procedure)
cancer_tests_CT_abdo	354951000000000	Computed tomography triple phase study of liver (procedure)
cancer_tests_CT_abdo	356261000000000	Computed tomography dual phase study of liver (procedure)
cancer_tests_CT_abdo	772201000000000	Computed tomography perfusion study of abdomen (procedure)
cancer_tests_CT_abdo	772121000000000	Computed tomography perfusion study of liver (procedure)
cancer_tests_CT_abdo	777291000000000	Computed tomography of head, neck, thorax and abdomen with contrast (procedure)
cancer_tests_CT_abdo	270541000000000	CT of abdomen
cancer_tests_CT_abdo	810081000000000	Computed tomography of head, neck, thorax and abdomen (procedure)
cancer_tests_CT_abdo	811261000000000	Computed tomography sinogram of abdomen (procedure)
cancer_tests_CT_abdo	942221000000000	Computerised tomography of abdomen for radiotherapy planning with contrast (procedure)
cancer_tests_CT_abdo	764738000	Percutaneous irreversible electroporation ablation of neoplasm of pancreas using computed tomography guidance (procedure)
cancer_tests_CT_abdo	764581000	Computed tomography nephrostography (procedure)
cancer_tests_CT_abdo	952571000000000	CT colonography
cancer_tests_CT_abdo	952561000000000	Computed tomography colonography (procedure)
cancer_tests_CT_abdo	430430003	Computed tomography of adrenal gland with contrast (procedure)
cancer_tests_CT_abdo	764580004	Computed tomography of stomach with contrast (procedure)
cancer_tests_CT_abdo	713558005	Single photon emission computed tomography using gallium 67 with computed tomography of abdomen (procedure)
cancer_tests_CT_abdo	713552006	Single photon emission computed tomography using iodine 123 meta-iodobenzylguanidine with computed tomography of abdomen (procedure)
cancer_tests_CT_abdo	443267007	Single photon emission computed tomography using dimercaptosuccinic acid with computed tomography of kidney (procedure)
cancer_tests_CT_abdo	443637005	Single photon emission computed tomography with computed tomography of hemangioma of liver (procedure)
cancer_tests_CT_abdo	726421000000000	Computed tomography of chest and abdomen (procedure)
cancer_tests_CT_abdo	443638000	Single photon emission computed tomography with computed tomography of liver and spleen (procedure)
cancer_tests_CT_abdo	726431000000000	Computed tomography of chest and abdomen
cancer_tests_CT_abdo	508111000000000	Single photon emission computed tomography with computed tomography of kidney using dimercaptosuccinic acid (procedure)
cancer_tests_CT_abdo	508131000000000	Single photon emission computed tomography with computed tomography of haemangioma of liver (procedure)
cancer_tests_CT_abdo	508141000000000	Single photon emission computed tomography with computed tomography of liver and spleen (procedure)
cancer_tests_CT_abdo	55501000	Computed tomography of kidney (procedure)
cancer_tests_CT_abdo	32962002	Computed tomography of abdomen with contrast (procedure)
cancer_tests_CT_abdo	1156740009	Percutaneous drainage of liver using computed tomography guidance (procedure)
cancer_tests_CT_abdo	1156745004	Percutaneous drainage of upper abdomen using computed tomography guidance (procedure)
cancer_tests_CT_abdo	1162472007	Biopsy of upper abdomen using computed tomography guidance (procedure)
cancer_tests_CT_abdo	314613007	Drainage of pancreatic lesion using computed tomography guidance (procedure)
cancer_tests_CT_abdo	1162474008	Drainage of pancreas using computed tomography guidance (procedure)
cancer_tests_CT_abdo	912341000000000	Computed tomography angiography of thorax and abdomen (procedure)
cancer_tests_CT_resultAbn	168791006	Neuroradiography with contrast abnormal (finding)
cancer_tests_CT_resultAbn	129679001	Computed tomography result abnormal (finding)
cancer_tests_CT_resultAbn	407670004	Computed tomography of brain abnormal (finding)
cancer_tests_CT_resultAbn	169030006	Tomography abnormal (finding)
cancer_tests_CT_resultAbn	169039007	Tomography - chest abnormal (finding)
cancer_tests_CT_resultAbn	1339044001	Computed tomography of nervous system with contrast abnormal (finding)
cancer_tests_CT_resultAbn	1268971002	Digital breast tomosynthesis abnormal (finding)
cancer_tests_CT_resultAbn	1343553005	Computed tomography of gallbladder with contrast abnormal (finding)
cancer_tests_CT_resultAbn	445398008	Computed tomography of head abnormal (finding)
cancer_tests_CT_resultAbn	324891000000000	Computerised tomography scan of brain abnormal (finding)
cancer_tests_CT_resultAbn	324901000000000	CT brain scan normal
cancer_tests_CT_resultAbn	324911000000000	CT brain scan normal
cancer_tests_CT_resultAbn	431914006	Computed tomography of brain abnormal (finding)
cancer_tests_CT_resultAbn	16900311000119102	Computed tomography of chest abnormal (finding)
cancer_tests_CT_resultAbn	15634181000119107	Computed tomography of abdomen abnormal (finding)
cancer_tests_CT_resultAbn	15634221000119104	Computed tomography of pelvis abnormal (finding)
cancer_tests_CT_resultAbn	15634081000119106	Computed tomography of biliary tract abnormal (finding)
cancer_tests_CT_resultAbn	15634131000119106	Computed tomography of abdominal wall abnormal (finding)

cancer_tests_USSabdo	710342007	Doppler ultrasonography of vein of abdomen (procedure)
cancer_tests_USSabdo	17001000000000	Puncture of cyst of left kidney using ultrasonographic guidance (procedure)
cancer_tests_USSabdo	183541000000000	US scan and aspiration of kidney (procedure)
cancer_tests_USSabdo	183201000000000	US scan and drainage of pancreas (procedure)
cancer_tests_USSabdo	183191000000000	US scan and biopsy of pancreas (procedure)
cancer_tests_USSabdo	183181000000000	US scan and aspiration of pancreas (procedure)
cancer_tests_USSabdo	181421000000000	US scan and drainage of liver (procedure)
cancer_tests_USSabdo	181411000000000	US scan and aspiration of liver (procedure)
cancer_tests_USSabdo	181401000000000	US scan and biopsy of liver (procedure)
cancer_tests_USSabdo	711279001	Doppler ultrasonography of vascular structure of intestine (procedure)
cancer_tests_USSabdo	711308006	Puncture of cyst of kidney using ultrasonographic guidance (procedure)
cancer_tests_USSabdo	13391000000000	Left nephrostomy using ultrasonographic guidance (procedure)
cancer_tests_USSabdo	13401000000000	Right nephrostomy using ultrasonographic guidance (procedure)
cancer_tests_USSabdo	17011000000000	Puncture of cyst of right kidney using ultrasonographic guidance (procedure)
cancer_tests_USSabdo	408071000000000	Percutaneous needle biopsy of kidney using ultrasonographic guidance (procedure)
cancer_tests_USSabdo	303916004	Hepatobiliary ultrasound (procedure)
cancer_tests_USSabdo	1264439009	Anastomosis of gallbladder to duodenum and insertion of stent using endoscopic ultrasonography guidance (procedure)
cancer_tests_USSabdo	14361000000000	Aspiration of cyst of bilateral kidneys using ultrasonographic guidance (procedure)
cancer_tests_USSabdo	16931000000000	Biopsy of bilateral adrenal glands using ultrasonographic guidance (procedure)
cancer_tests_USSabdo	314615000	Aspiration of pancreatic lesion using ultrasonic guidance (procedure)
cancer_tests_USSabdo	13871000000000	Aspiration of cyst of left kidney using ultrasonographic guidance (procedure)
cancer_tests_USSabdo	13861000000000	Aspiration of cyst of right kidney using ultrasonographic guidance (procedure)
cancer_tests_USSabdo	1151000000000	Biopsy of right kidney using ultrasonographic guidance (procedure)
cancer_tests_USSabdo	1141000000000	Biopsy of left kidney using ultrasonographic guidance (procedure)
cancer_tests_USSabdo	723978006	Biopsy of adrenal gland using ultrasonographic guidance (procedure)
cancer_tests_USSabdo	11931000000000	Biopsy of left adrenal gland using ultrasonographic guidance (procedure)
cancer_tests_USSabdo	12191000000000	Biopsy of right adrenal gland using ultrasonographic guidance (procedure)
cancer_tests_USSabdo	711424006	Cholecystostomy using ultrasonographic guidance (procedure)
cancer_tests_USSabdo	711504009	Doppler ultrasonography of transjugular intrahepatic portosystemic shunt stent (procedure)
cancer_tests_USSabdo	1264335004	Gastrojejunostomy and insertion of stent using endoscopic ultrasonography guidance (procedure)
cancer_tests_USSabdo	441491005	Ultrasound elastography of liver (procedure)
cancer_tests_USSabdo	31683008	Retroperitoneal echography, B-scan, complete (procedure)
cancer_tests_USSabdo	277668001	Ultrasound scan of pancreas (procedure)
cancer_tests_USSabdo	1044001	Diagnostic ultrasound of abdomen and retroperitoneum (procedure)
cancer_tests_USSabdo	175951002	Endoscopic ultrasound fragmentation of renal calculus (procedure)
cancer_tests_USSabdo	880064002	Fine needle aspiration of retroperitoneal lymph node using endoscopic ultrasonography guidance (procedure)
cancer_tests_USSabdo	306005	Echography of kidney (procedure)
cancer_tests_USSabdo	442993000	Percutaneous radiofrequency ablation of lesion of liver using ultrasound guidance (procedure)
cancer_tests_USSabdo	709639005	Doppler ultrasonography of portal vein (procedure)
cancer_tests_USSabdo	709643009	Biopsy of peritoneum using ultrasonographic guidance (procedure)
cancer_tests_USSabdo	709640007	Doppler ultrasonography of renal vein (procedure)
cancer_tests_USSabdo	709642004	Biopsy of retroperitoneum using ultrasonographic guidance (procedure)
cancer_tests_USSabdo	709644003	Biopsy of spleen using ultrasonographic guidance (procedure)
cancer_tests_USSabdo	184601000000000	US scan and drainage of upper abdomen (procedure)
cancer_tests_USSabdo	990271000000000	Ultrasonography surveillance of liver for hepatocellular carcinoma (procedure)
cancer_tests_USSabdo	184591000000000	US scan and biopsy of upper abdomen (procedure)
cancer_tests_USSabdo	184491000000000	US scan of lower abdomen (procedure)
cancer_tests_USSabdo	865581000000000	Endoscopic brush biopsy of lesion of pancreas using ultrasonography guidance (procedure)
cancer_tests_USSabdo	859251000000000	Ultrasonography of small intestine (procedure)
cancer_tests_USSabdo	184481000000000	US scan of upper abdomen (procedure)
cancer_tests_USSabdo	442887009	Percutaneous needle biopsy of pancreas using ultrasound guidance (procedure)
cancer_tests_USSabdo	78579002	Ultrasonography of retroperitoneum (procedure)
cancer_tests_USSabdo	184451000000000	US scan of diaphragm (procedure)
cancer_tests_USSabdo	184401000000000	US scan of abdominal vessels (procedure)
cancer_tests_USSabdo	184391000000000	US scan of abdomen and pelvis (procedure)
cancer_tests_USSabdo	184351000000000	US doppler scan of kidney (procedure)
cancer_tests_USSabdo	853781000000000	Percutaneous insertion of peritoneal-amniotic shunt using ultrasonography guidance (procedure)
cancer_tests_USSabdo	851041000000000	Endoscopic examination of pancreatic duct using ultrasonography guidance (procedure)
cancer_tests_USSabdo	850871000000000	Endoscopic fine needle aspiration biopsy of pancreas using ultrasonography guidance (procedure)
cancer_tests_USSabdo	849951000000000	Endoscopic core needle biopsy of pancreas using ultrasonography guidance (procedure)
cancer_tests_USSabdo	849901000000000	Endoscopic core needle biopsy of duodenum using ultrasonography guidance (procedure)
cancer_tests_USSabdo	710341000	Doppler ultrasonography of vascular structure of abdomen (procedure)
cancer_tests_USSabdo	849891000000000	Endoscopic fine needle aspiration biopsy of duodenum using ultrasonography guidance (procedure)
cancer_tests_USSabdo	849871000000000	Endoscopic core needle biopsy of stomach using ultrasonography guidance (procedure)
cancer_tests_USSabdo	849861000000000	Endoscopic fine needle aspiration biopsy of stomach using ultrasonography guidance (procedure)
cancer_tests_USSabdo	849711000000000	Endoscopic ultrasonography of duodenal ampulla (procedure)
cancer_tests_USSabdo	710197007	Ultrasonography of appendix (procedure)
cancer_tests_USSabdo	184321000000000	US scan and aspiration of upper abdomen (procedure)
cancer_tests_USSabdo	183551000000000	US scan and biopsy of kidney (procedure)
cancer_tests_USSabdo	184071000000000	US angiography of renal artery (procedure)
cancer_tests_USSabdo	16451621000119103	Needle biopsy of left kidney using ultrasonographic guidance (procedure)
cancer_tests_USSabdo	16451571000119101	Needle biopsy of right kidney using ultrasonographic guidance (procedure)
cancer_tests_USSabdo	16443041000119100	Percutaneous needle biopsy of liver using ultrasonographic guidance (procedure)
cancer_tests_USSabdo	183561000000000	US scan and drainage of kidney (procedure)
cancer_tests_USSabdo	718078008	Doppler ultrasonography of transplanted liver (procedure)
cancer_tests_USSabdo	517751000000000	Ultrasonography of abdomen with contrast (procedure)
cancer_tests_USSabdo	320991000000000	Ultrasound guided drainage of kidney cyst (procedure)
cancer_tests_USSabdo	715966005	Aspiration of spleen using ultrasonographic guidance (procedure)
cancer_tests_USSabdo	702690005	Doppler ultrasound of transplant liver (procedure)
cancer_tests_USSabdo	320981000000000	Ultrasound guided ablation of kidney cyst (procedure)
cancer_tests_USSabdo	718290002	Ultrasonography of liver for early detection of hepatocellular carcinoma (procedure)
cancer_tests_USSabdo	1284923001	Drainage of gallbladder using endoscopic ultrasonography guidance (procedure)
cancer_tests_USSabdo	702594008	Ultrasound guided needle localization of abdomen (procedure)
cancer_tests_USSabdo	702585002	Doppler ultrasonography of renal artery (procedure)
cancer_tests_USSabdo	314271000000000	Ultrasound doppler scan of superior mesenteric vein (procedure)
cancer_tests_USSabdo	314261000000000	Ultrasound doppler scan of superior mesenteric artery (procedure)
cancer_tests_USSabdo	313631000000000	Ultrasound scan of anterior abdominal wall (procedure)
cancer_tests_USSabdo	703869007	Drainage of abdomen using ultrasound guidance (procedure)
cancer_tests_USSabdo	312981000000000	Ultrasound doppler scan of liver and portal system (procedure)
cancer_tests_USSabdo	310341000000000	Endoscopic ultrasound guided aspiration of pancreas (procedure)
cancer_tests_USSabdo	310321000000000	Ultrasound guided aspiration of abdomen (procedure)
cancer_tests_USSabdo	48996003	Echography of abdomen, B-scan, limited (procedure)
cancer_tests_USSabdo	480571000000000	Doppler ultrasound velocimetry of umbilical artery of fetus (procedure)
cancer_tests_USSabdo	16460061000119103	Percutaneous needle biopsy of left kidney using ultrasonographic guidance (procedure)
cancer_tests_USSabdo	16460111000119102	Percutaneous needle biopsy of right kidney using ultrasonographic guidance (procedure)
cancer_tests_USSabdo	310211000000000	Endoscopic ultrasound examination of stomach (procedure)
cancer_tests_USSabdo	310191000000000	Ultrasound guided drainage of abdomen (procedure)
cancer_tests_USSabdo	16446101000119108	Ultrasonography of abdomen for ascites (procedure)

cancer_tests_USSabdo	756151000000000	Ultrasound elastography of liver (procedure)
cancer_tests_USSabdo	758221000000000	Ultrasound elastography of liver
cancer_tests_USSabdo	808781000000000	US scan of abdominal aorta
cancer_tests_USSabdo	927521000000000	Ultrasonography of spleen with contrast (procedure)
cancer_tests_USSabdo	426701000000000	Ultrasonography of abdomen, right upper quadrant and epigastrium (procedure)
cancer_tests_USSabdo	1076691000000108	Three dimensional ultrasonography of abdominal aortic endovascular aneurysm repair with contrast (procedure)
cancer_tests_USSabdo	419739000	Ultrasound scan and biopsy of liver (procedure)
cancer_tests_USSabdo	449348003	Endoscopic ultrasonography of retroperitoneum (procedure)
cancer_tests_USSabdo	448587007	Aspiration of cyst of kidney using ultrasound guidance (procedure)
cancer_tests_USSabdo	418276008	Ultrasound scan and aspiration of liver (procedure)
cancer_tests_USSabdo	897715002	Fine needle aspiration of abdominal lymph node using endoscopic ultrasonography guidance (procedure)
cancer_tests_USSabdo	715968006	Doppler ultrasonography of renal vessel (procedure)
cancer_tests_USSabdo	417958008	Ultrasound scan and drainage of liver (procedure)
cancer_tests_USSabdo	241496002	Fetal echocardiography (procedure)
cancer_tests_USSabdo	418705007	Ultrasound scan and aspiration of pancreas (procedure)
cancer_tests_USSabdo	449061009	Endoscopic ultrasonography and biopsy of retroperitoneum (procedure)
cancer_tests_USSabdo	418400003	Ultrasound scan and biopsy of pancreas (procedure)
cancer_tests_USSabdo	129120004	Ultrasound phlebography of hepatic vein (procedure)
cancer_tests_USSabdo	420007006	Ultrasound scan and drainage of pancreas (procedure)
cancer_tests_USSabdo	418354002	Doppler ultrasonography of kidney (procedure)
cancer_tests_USSabdo	418704006	Ultrasound scan and aspiration of kidney (procedure)
cancer_tests_USSabdo	419174006	Ultrasound scan and biopsy of kidney (procedure)
cancer_tests_USSabdo	419467008	Ultrasound scan and drainage of kidney (procedure)
cancer_tests_USSabdo	418231008	Ultrasound angiography of renal artery (procedure)
cancer_tests_USSabdo	882571000000000	Obstetric ultrasonography of ductus venosus (procedure)
cancer_tests_USSabdo	881061000000000	Doppler ultrasonography of superior epigastric artery (procedure)
cancer_tests_USSabdo	716518003	Fine needle aspiration biopsy of liver using ultrasonographic guidance (procedure)
cancer_tests_USSabdo	1197441007	Ultrasonography of kidney with contrast (procedure)
cancer_tests_USSabdo	716554004	Removal of tunneled peritoneal drain from peritoneal cavity using ultrasonographic guidance (procedure)
cancer_tests_USSabdo	716558001	Insertion of tunneled peritoneal drain into peritoneal cavity using ultrasonographic guidance (procedure)
cancer_tests_USSabdo	794701000000000	Ultrasound scan of abdomen and pelvis
cancer_tests_USSabdo	45036003	Ultrasonography of abdomen (procedure)
cancer_tests_USSabdo	241474008	Ultrasound scan of transplant pancreas (procedure)
cancer_tests_USSabdo	241475009	Ultrasound scan of pylorus (procedure)
cancer_tests_USSabdo	241476005	Ultrasound scan of spleen (procedure)
cancer_tests_USSabdo	794691000000000	Ultrasonography of abdomen and pelvis (procedure)
cancer_tests_USSabdo	241477001	Ultrasound scan of bowel (procedure)
cancer_tests_USSabdo	241478006	Ultrasound scan of psoas (procedure)
cancer_tests_USSabdo	792211000000000	Ultrasound scan of upper abdomen
cancer_tests_USSabdo	792201000000000	Ultrasound scan of upper abdomen (procedure)
cancer_tests_USSabdo	241480000	Ultrasound scan of adrenals (procedure)
cancer_tests_USSabdo	241481001	Ultrasound scan of kidney and ureters (procedure)
cancer_tests_USSabdo	235381000000000	Obstetric umbilical artery Doppler (procedure)
cancer_tests_USSabdo	233091000000000	Laparoscopic ultrasound examination of liver NEC (procedure)
cancer_tests_USSabdo	792161000000000	Ultrasound scan of lower abdomen
cancer_tests_USSabdo	792151000000000	Ultrasound scan of lower abdomen (procedure)
cancer_tests_USSabdo	241479003	Subphrenic ultrasound scan (procedure)
cancer_tests_USSabdo	785081000000000	Nephrostomy using ultrasound guidance (procedure)
cancer_tests_USSabdo	431363000	Doppler ultrasonography of superior mesenteric artery (procedure)
cancer_tests_USSabdo	1332562000	Balloon dilation of nephrostomy using ultrasonographic guidance (procedure)
cancer_tests_USSabdo	450460004	Nephrostomy using ultrasound guidance (procedure)
cancer_tests_USSabdo	1641000000000	Biopsy of bilateral kidneys using ultrasonographic guidance (procedure)
cancer_tests_USSabdo	979441000000000	Insertion of tunnelled drain into abdomen using ultrasonographic guidance (procedure)
cancer_tests_USSabdo	979421000000000	Removal of tunnelled drain from abdomen using ultrasonographic guidance (procedure)
cancer_tests_USSabdo	974761000000000	Ultrasonography of gallbladder with contrast (procedure)
cancer_tests_USSabdo	963461000000000	Fine needle aspiration biopsy of liver using ultrasonographic guidance (procedure)
cancer_tests_USSabdo	866202009	Ultrasonography of abdominal wall (procedure)
cancer_tests_USSabdo	866199004	Ultrasonography of intestine with contrast (procedure)
cancer_tests_USSabdo	44052005	Ultrasonography of biliary tract (procedure)
cancer_tests_USSabdo	169250003	Ultrasound scan of gallbladder (procedure)
cancer_tests_USSabdo	241473002	Ultrasound scan of native pancreas (procedure)
cancer_tests_USSabdo	241472007	Ultrasound scan of transplant liver (procedure)
cancer_tests_USSabdo	241471000	Ultrasound scan of native liver (procedure)
cancer_tests_USSabdo	241462009	Ultrasound scan of abdominal aorta (procedure)
cancer_tests_USSabdo	1111711000000104	Injection into scar tissue of anterior abdominal wall using ultrasonographic guidance (procedure)
cancer_tests_USSabdo	1111761000000102	Point of care ultrasonography of abdomen (procedure)
cancer_tests_USSabdo	1853651000000105	Shear wave elastography ultrasonography of liver (procedure)
cancer_tests_USSabdo	419528000	Ultrasound scan and drainage of upper abdomen (procedure)
cancer_tests_USSabdo	419257000	Ultrasound scan and biopsy of upper abdomen (procedure)
cancer_tests_USSabdo	420052009	Ultrasound scan of lower abdomen (procedure)
cancer_tests_USSabdo	1089921000000103	Intraoperative ultrasonography of abdomen (procedure)
cancer_tests_USSabdo	418398002	Ultrasound scan of upper abdomen (procedure)
cancer_tests_USSabdo	418889006	Ultrasound scan of diaphragm (procedure)
cancer_tests_USSabdo	418733007	Ultrasound scan of abdominal vessels (procedure)
cancer_tests_USSabdo	418394000	Ultrasound scan of abdomen and pelvis (procedure)
cancer_tests_USSabdo	418474008	Ultrasound scan and aspiration of upper abdomen (procedure)
cancer_tests_USSabdo	237211000000000	Endoscopic ultrasound examination of bile duct and biopsy of lesion of bile duct (procedure)
cancer_tests_USSabdo	247751000000000	Laparoscopic ultrasound of liver (procedure)
cancer_tests_USSabdo	440499003	Radiofrequency ablation of lesion of kidney using ultrasound guidance (procedure)
cancer_tests_USSabdo	438300009	Biopsy of liver using ultrasound guidance (procedure)
cancer_tests_USSabdo	438567008	Intrauterine fetal transfusion using ultrasound guidance (procedure)
cancer_tests_USSabdo	440452005	Percutaneous aspiration of pancreas using ultrasound guidance (procedure)
cancer_tests_USSabdo	439864002	Focused assessment with ultrasonography for trauma (procedure)
cancer_tests_USSabdo	438416007	Ultrasonography of abdomen and urinary system (procedure)
cancer_tests_USSabdo	438345008	Aspiration of kidney using ultrasound guidance (procedure)
cancer_tests_USSabdo	438815000	Radiofrequency ablation of lesion of liver using ultrasound guidance (procedure)
cancer_tests_USSabdo	438813007	Biopsy of kidney using ultrasound guidance (procedure)
cancer_tests_USSabdo	71163001	Echography of abdomen, B-scan, complete (procedure)
cancer_tests_USSabdo	353031000000000	Ultrasonography of abdomen and renal tract (procedure)
cancer_tests_USSabdo	350351000000000	Percutaneous aspiration of pancreas using ultrasound guidance (procedure)
cancer_tests_USSabdo	350311000000000	Biopsy of liver using ultrasound guidance (procedure)
cancer_tests_USSabdo	350301000000000	Biopsy of kidney using ultrasound guidance (procedure)
cancer_tests_USSabdo	13301000000000	Ultrasonography of bilateral kidneys (procedure)
cancer_tests_USSabdo	699579008	Doppler ultrasonography of superior epigastric artery (procedure)
cancer_tests_USSabdo	427108001	Endoscopic ultrasound examination of liver (procedure)
cancer_tests_USSabdo	11581000000000	Ultrasonography of left kidney (procedure)
cancer_tests_USSabdo	446886003	Endoscopic ultrasonography of small intestine (procedure)
cancer_tests_USSabdo	426859009	Endoscopic ultrasound examination of bile duct (procedure)

cancer_tests_USSabdo	11521000000000	Ultrasonography of right kidney (procedure)
cancer_tests_USSabdo	426808008	Endoscopic ultrasound examination of pancreas (procedure)
cancer_tests_USSabdo	11390003	Retroperitoneal echography, B-scan, limited (procedure)
cancer_tests_USSabdo	425876002	Endoscopic ultrasound examination of pancreas and biopsy of lesion of pancreas (procedure)
cancer_tests_USSabdo	426225003	Diagnostic endoscopic ultrasound examination of peritoneum (procedure)
cancer_tests_USSabdo	11681000000000	Ultrasonography of right adrenal gland (procedure)
cancer_tests_USSabdo	427703003	Diagnostic endoscopic ultrasound examination of peritoneum and biopsy of intraabdominal organ (procedure)
cancer_tests_USSabdo	11651000000000	Ultrasonography of bilateral adrenal glands (procedure)
cancer_tests_USSabdo	11691000000000	Ultrasonography of left adrenal gland (procedure)
cancer_tests_USSabdo	427112007	Endoscopic ultrasound examination of bile duct and biopsy of lesion of bile duct (procedure)
cancer_tests_USSabdo	427623005	Obstetric umbilical artery Doppler (procedure)
cancer_tests_USSabdo	840542000	Measurement of controlled attenuation parameter (procedure)
cancer_tests_USSabdo	449036006	Endoscopic ultrasonography and biopsy of bile duct (procedure)
cancer_tests_USSabdo	314614001	Ultrasound guided drainage of pancreatic lesion (procedure)
cancer_tests_USSabdo	314618003	Operative abdominal ultrasound examination (procedure)
cancer_tests_USSabdo	446595004	Biopsy of abdomen using ultrasound guidance (procedure)
cancer_tests_USSabdo	433221009	Celiac plexus block using ultrasound guidance (procedure)
cancer_tests_USSabdo	432582008	Drainage of cyst of kidney using ultrasound guidance (procedure)
cancer_tests_USSabdo	446651002	Upper endoscopic ultrasonography of retroperitoneum (procedure)
cancer_tests_USSabdo	1336127004	Percutaneous transhepatic insertion of catheter into biliary tract using ultrasonographic guidance (procedure)
cancer_tests_USSabdo	1268385000	Anastomosis of hepatic duct to stomach and insertion of biliary stent using endoscopic ultrasonography guidance (procedure)
cancer_tests_USSabdo	1268399001	Cystogastrostomy of pancreas and insertion of stent using endoscopic ultrasonography guidance (procedure)
cancer_tests_USSabdo	444900008	Ultrasonography of abdomen with contrast (procedure)
cancer_tests_USSabdo	446012006	Endoscopic ultrasonography and biopsy of stomach (procedure)
cancer_tests_USSabdo	1268402000	Choledochoduodenostomy and insertion of biliary stent using endoscopic ultrasonography guidance (procedure)
cancer_tests_USSabdo	446011004	Endoscopic ultrasonography of colon (procedure)
cancer_tests_USSabdo	431894006	Ultrasonography of transjugular intrahepatic portosystemic shunt stent (procedure)
cancer_tests_USSabdo	432422008	Ablation of cyst of kidney using ultrasound guidance (procedure)
cancer_tests_USSabdo	446010003	Endoscopic ultrasonography and biopsy of colon (procedure)
cancer_tests_USSabdo	446542002	Endoscopic destruction of celiac plexus using ultrasound guidance (procedure)
cancer_tests_USSabdo	17375006	Ultrasonic guidance for renal pelvis aspiration (procedure)
cancer_tests_USSabdo	432543006	Endoscopic aspiration of pancreas using ultrasound guidance (procedure)
cancer_tests_USSabdo	700442004	Ultrasonography of fetal ductus venosus (procedure)
cancer_tests_USSabdo	715193009	Ultrasonography of gallbladder with contrast (procedure)
cancer_tests_USSabdo	315289004	Ultrasound guided drainage of abdominal abscess (procedure)
cancer_tests_USSabdo	709420008	Endoscopic ultrasonography of biliary tract (procedure)
cancer_tests_USSabdo	443630007	Ultrasonography of liver with contrast (procedure)
cancer_tests_USSabdo	1216947003	Anastomosis of hepatic duct to stomach using endoscopic ultrasonography guidance (procedure)
cancer_tests_USSabdo	105377009	Ultrasonography of liver (procedure)
cancer_tests_USSabdo	911511000000000	Doppler ultrasonography of transplanted liver (procedure)
cancer_tests_USSabdo	911671000000000	Biopsy of transplanted liver using ultrasonography guidance (procedure)
cancer_tests_USSabdo	1216987008	Choledochoduodenostomy using endoscopic ultrasonography guidance (procedure)
cancer_tests_USSabdo	443755009	Percutaneous biopsy of liver using ultrasound guidance (procedure)
cancer_tests_USSabdo	1217311004	Biopsy of bile duct using endoscopic ultrasonography guidance (procedure)
cancer_tests_USSabdo	1217313001	Biopsy of colon using endoscopic ultrasonography guidance (procedure)
cancer_tests_USSabdo	1217314007	Biopsy of lesion of bile duct using endoscopic ultrasonography guidance (procedure)
cancer_tests_USSabdo	1217396008	Biopsy of upper abdomen using ultrasonographic guidance (procedure)
cancer_tests_USSabdo	1217397004	Biopsy of pancreas using ultrasonographic guidance (procedure)
cancer_tests_USSabdo	1217420009	Biopsy of lesion of liver using endoscopic ultrasonography guidance (procedure)
cancer_tests_USSabdo	1217422001	Biopsy of lesion of pancreas using endoscopic ultrasonography guidance (procedure)
cancer_tests_USSabdo	1220539004	Endoscopic ultrasonography of peritoneum (procedure)
cancer_tests_USSabdo	1220540002	Endoscopic ultrasonography of peritoneum and biopsy of intraabdominal organ (procedure)
cancer_tests_USSabdo	1220578000	Biopsy of stomach using endoscopic ultrasonography guidance (procedure)
cancer_tests_USSabdo	1220579008	Biopsy of retroperitoneum using endoscopic ultrasonography guidance (procedure)
cancer_tests_USSabdo	1220605009	Endoscopic ultrasonography of peritoneum and biopsy of lesion of peritoneum (procedure)
cancer_tests_USSabdo	1300176002	Drainage of biliary tract using endoscopic ultrasonography guidance (procedure)
cancer_tests_USSabdo	442833003	Percutaneous cholecystostomy and drainage using ultrasound guidance (procedure)
cancer_tests_USSabdo	442876001	Percutaneous drainage of retroperitoneal abscess using ultrasound guidance (procedure)
cancer_tests_USSabdo	948381000000000	Biopsy of spleen using ultrasonographic guidance (procedure)
cancer_tests_USSabdo	433043005	Insertion of drain into abdomen using ultrasound guidance (procedure)
cancer_tests_USSabdo	432335000	Doppler ultrasonography of superior mesenteric vein (procedure)
cancer_tests_USSabdo	432853001	Ultrasonography of anterior abdominal wall (procedure)
cancer_tests_USSabdo	257921000000000	Laparoscopic ultrasound examination of liver NEC
cancer_tests_USSabdo	258171000000000	Endoscopic ultrasound examination of liver
cancer_tests_USSabdo	258181000000000	Endoscopic ultrasound examination of liver and biopsy of lesion of liver
cancer_tests_USSabdo	258191000000000	Other specified endoscopic ultrasound examination of liver
cancer_tests_USSabdo	258201000000000	Endoscopic ultrasound examination of liver NOS
cancer_tests_USSabdo	258401000000000	Other specified laparoscopic ultrasound examination of bile duct
cancer_tests_USSabdo	258421000000000	Endoscopic ultrasound examination of bile duct
cancer_tests_USSabdo	258431000000000	Endoscopic ultrasound examination of bile duct and biopsy of lesion of bile duct
cancer_tests_USSabdo	773321000000000	Aspiration of cyst of kidney using ultrasound guidance (procedure)
cancer_tests_USSabdo	258441000000000	Other specified endoscopic ultrasound examination of bile duct
cancer_tests_USSabdo	258451000000000	Endoscopic ultrasound examination of bile duct NOS
cancer_tests_USSabdo	258521000000000	Other specified laparoscopic ultrasound examination of pancreas
cancer_tests_USSabdo	258541000000000	Endoscopic ultrasound examination of pancreas
cancer_tests_USSabdo	258551000000000	Endoscopic ultrasound examination of pancreas and biopsy of lesion of pancreas
cancer_tests_USSabdo	258561000000000	Other specified endoscopic ultrasound examination of pancreas
cancer_tests_USSabdo	258571000000000	Endoscopic ultrasound examination of pancreas NOS
cancer_tests_USSabdo	263901000000000	Obstetric umbilical artery Doppler
cancer_tests_USSabdo	264281000000000	Diagnostic endoscopic ultrasound examination of peritoneum
cancer_tests_USSabdo	264291000000000	Diagnostic endoscopic ultrasound examination of peritoneum and biopsy of intraabdominal organ
cancer_tests_USSabdo	270551000000000	US scan of abdomen
cancer_tests_USSabdo	270891000000000	Renal ultrasound
cancer_tests_USSabdo	378391000000000	Ultrasound elastography of liver (procedure)
cancer_tests_USSabdo	431362005	Doppler ultrasonography of liver and portal system (procedure)
cancer_tests_USSabdo	431841002	Endoscopic ultrasonography of stomach (procedure)
cancer_tests_USSabdo	432556004	Aspiration of abdomen using ultrasound guidance (procedure)
cancer_tests_USSabdo	224901000000000	Diagnostic endoscopic ultrasound examination of peritoneum and biopsy of intraabdominal organ (procedure)
cancer_tests_USSabdo	224891000000000	Diagnostic endoscopic ultrasound examination of peritoneum (procedure)
cancer_tests_USSabdo	905871000000000	Insertion of nephrostomy tube into transplanted kidney using ultrasonography guidance (procedure)
cancer_tests_USSabdo	264972006	Diagnostic endoscopic examination of peritoneum and biopsy of lesion of peritoneum (procedure)
cancer_tests_USSabdo	788641000	Fine needle aspiration of lesion of pancreas using endoscopic ultrasonography guidance (procedure)
cancer_tests_USSabdo	334481000000000	Ultrasound guidance for coeliac plexus block (procedure)
cancer_tests_USSabdo	1231314009	Cystogastrostomy of pancreas using endoscopic ultrasonography guidance (procedure)
cancer_tests_USSabdo	900031000000000	Ultrasonography of kidney with contrast (procedure)
cancer_tests_USSabdo	334871000000000	Ultrasound scan check of transjugular intrahepatic portosystemic shunt stent (procedure)
cancer_tests_USSabdo	788639001	Insertion of fiducial marker into pancreas using endoscopic ultrasonography guidance (procedure)
cancer_tests_USSabdo	764971006	Three dimensional ultrasonography of abdominal aortic endovascular aneurysm repair with contrast (procedure)

Abdominal Scans: Ultrasound

cancer_tests_USSabdo	224381000000000	Endoscopic ultrasound examination of pancreas NOS (procedure)
cancer_tests_USSabdo	224271000000000	Other specified endoscopic ultrasound examination of pancreas (procedure)
cancer_tests_USSabdo	10321000000000	Ultrasonography of chest and abdomen (procedure)
cancer_tests_USSabdo	36960008	Intra-abdominal phlebography (procedure)
cancer_tests_USSabdo	223711000000000	Endoscopic ultrasound examination of liver NOS (procedure)
cancer_tests_USSabdo	224261000000000	Endoscopic ultrasound examination of pancreas and biopsy of lesion of pancreas (procedure)
cancer_tests_USSabdo	339271000000000	Aspiration of kidney using ultrasound guidance (procedure)
cancer_tests_USSabdo	508511000000000	Ultrasonography of liver with contrast (procedure)
cancer_tests_USSabdo	223801000000000	Endoscopic ultrasound examination of bile duct NOS (procedure)
cancer_tests_USSabdo	223821000000000	Endoscopic ultrasound examination of liver (procedure)
cancer_tests_USSabdo	224251000000000	Endoscopic ultrasound examination of pancreas (procedure)
cancer_tests_USSabdo	223861000000000	Other specified laparoscopic ultrasound examination of bile duct (procedure)
cancer_tests_USSabdo	224241000000000	Laparoscopic ultrasound examination of pancreas NOS (procedure)
cancer_tests_USSabdo	339301000000000	Radiofrequency ablation of kidney lesion using ultrasound guidance (procedure)
cancer_tests_USSabdo	750261000000000	Biopsy of abdomen using ultrasonographic guidance (procedure)
cancer_tests_USSabdo	223871000000000	Laparoscopic ultrasound examination of bile duct NOS (procedure)
cancer_tests_USSabdo	712868000	Puncture and aspiration of abdomen using ultrasonographic guidance (procedure)
cancer_tests_USSabdo	339321000000000	Radiofrequency ablation of liver lesion using ultrasound guidance (procedure)
cancer_tests_USSabdo	713495007	Ultrasonography of spleen with contrast (procedure)
cancer_tests_USSabdo	223881000000000	Endoscopic ultrasound examination of bile duct (procedure)
cancer_tests_USSabdo	223981000000000	Other specified endoscopic ultrasound examination of liver (procedure)
cancer_tests_USSabdo	224231000000000	Other specified laparoscopic ultrasound examination of pancreas (procedure)
cancer_tests_USSabdo	1287681009	Transabdominal ultrasonography of intestine (procedure)
cancer_tests_USSabdo	223991000000000	Endoscopic ultrasound examination of liver and biopsy of lesion of liver (procedure)
cancer_tests_USSabdo	224091000000000	Other specified endoscopic ultrasound examination of bile duct (procedure)
cancer_tests_USSabdoJS	202541000000000	Renal ultrasound abnormal (finding)
cancer_tests_USSabdoJS	890401000000000	Renal ultrasound abnormal
cancer_tests_USSabdoJS	15633881000119102	Ultrasonography of liver abnormal (finding)
cancer_tests_USSabdoJS	809101000000000	Abdominal aortic aneurysm screen ultrasound scan abnormal (finding)
cancer_tests_USSabdoJS	809111000000000	Abdominal aortic aneurysm ultrasound scan abnormal

Cancer Referrals

cancer_2ww_referrals_UpperGI	276561000000000	Urgent cancer referral - upper gastrointestinal
cancer_2ww_referrals_UpperGI	276551000000000	Fast track referral for suspected upper gastrointestinal cancer (procedure)
cancer_2ww_referrals_UpperGI	276571000000000	Urgent cancer referral - upper gastrointestinal
referrals_2ww_UpperGI_seen	382831000000000	Seen in fast track suspected upper gastrointestinal cancer clinic
referrals_2ww_UpperGI_seen	382821000000000	Seen in fast track suspected upper gastrointestinal cancer clinic
referrals_2ww_UpperGI_seen	382811000000000	Seen in fast track suspected upper gastrointestinal cancer clinic (finding)
cancer_2ww_referrals_	288261000000000	Fast track cancer referral
cancer_2ww_referrals_	292461000000000	Fast track cancer referral
cancer_2ww_referrals_	1556551000000104	Fast track referral for suspected cancer due to non-site-specific symptoms (procedure)
cancer_2ww_referrals_	199251000000000	Fast track cancer referral (procedure)
cancer_2ww_referrals_	892211000000000	Fast track referral for suspected lower GI cancer
cancer_2ww_referrals_	892201000000000	Fast track referral for suspected lower gastrointestinal cancer (procedure)
cancer_2ww_referrals_	214681000000000	Fast track cancer referral
cancer_2ww_referrals_	214671000000000	Fast track cancer referral
cancer_2ww_referrals_	276571000000000	Urgent cancer referral - upper gastrointestinal
cancer_2ww_referrals_	276601000000000	Urgent cancer referral - brain
cancer_2ww_referrals_	276581000000000	Fast track referral for suspected brain tumour (procedure)
cancer_2ww_referrals_	280521000000000	Fast track referral for suspected head and neck cancer (procedure)
cancer_2ww_referrals_	276591000000000	Urgent cancer referral - brain
cancer_2ww_referrals_	280531000000000	Fast track referral for head and neck cancer
cancer_2ww_referrals_	280541000000000	Fast track referral for head and neck cancer
cancer_2ww_referrals_	276561000000000	Urgent cancer referral - upper gastrointestinal
cancer_2ww_referrals_	276551000000000	Fast track referral for suspected upper gastrointestinal cancer (procedure)
cancer_2ww_referrals_	276281000000000	Fast track referral for suspected skin cancer (procedure)
cancer_2ww_referrals_	276541000000000	Urgent cancer referral - sarcoma
cancer_2ww_referrals_	276531000000000	Urgent cancer referral - sarcoma
cancer_2ww_referrals_	276291000000000	Urgent cancer referral - skin
cancer_2ww_referrals_	276301000000000	Urgent cancer referral - skin
cancer_2ww_referrals_	276311000000000	Fast track referral for suspected gynaecological cancer (procedure)
cancer_2ww_referrals_	276521000000000	Fast track referral for suspected sarcoma (procedure)
cancer_2ww_referrals_	276511000000000	Urgent cancer referral - lung
cancer_2ww_referrals_	276501000000000	Urgent cancer referral - lung
cancer_2ww_referrals_	276491000000000	Fast track referral for suspected lung cancer (procedure)
cancer_2ww_referrals_	276321000000000	Urgent cancer referral - gynaecology
cancer_2ww_referrals_	276331000000000	Urgent cancer referral - gynaecology
cancer_2ww_referrals_	276481000000000	Urgent cancer referral - haematology
cancer_2ww_referrals_	276471000000000	Urgent cancer referral - haematology
cancer_2ww_referrals_	276461000000000	Fast track referral for suspected haematology malignancy (procedure)
cancer_2ww_referrals_	276451000000000	Urgent cancer referral - urology
cancer_2ww_referrals_	276441000000000	Urgent cancer referral - urology
cancer_2ww_referrals_	276431000000000	Fast track referral for suspected urological cancer (procedure)
cancer_2ww_referrals_	276421000000000	Urgent cancer referral - colorectal
cancer_2ww_referrals_	276411000000000	Urgent cancer referral - colorectal
cancer_2ww_referrals_	276401000000000	Fast track referral for suspected colorectal cancer (procedure)
cancer_2ww_referrals_	276391000000000	Urgent cancer referral - children
cancer_2ww_referrals_	276381000000000	Urgent cancer referral - children
cancer_2ww_referrals_	276371000000000	Fast track referral for suspected childrens tumour (procedure)
cancer_2ww_referrals_	276361000000000	Urgent cancer referral - breast
cancer_2ww_referrals_	276341000000000	Fast track referral for suspected breast cancer (procedure)
cancer_2ww_referrals_	276351000000000	Urgent cancer referral - breast

Weight Loss

weight_loss	139911003	O/E - cachexic (finding)
weight_loss	496901000000000	[D]Abnormal loss of weight (situation)
weight_loss	501901000000000	[D]Cachexia (situation)
weight_loss	448765001	Unintentional weight loss (finding)
weight_loss	139089007	Weight decreasing (finding)
weight_loss	161834000	Abnormal weight loss (& [symptom]) (finding)
weight_loss	267158006	Weight loss (& abnormal) (finding)
weight_loss	430237002	Abnormal intentional weight loss (finding)
weight_loss	198511000000000	Complaining of weight loss (finding)
weight_loss	213801000000000	Complaining of weight loss
weight_loss	213791000000000	Complaining of weight loss
weight_loss	206919000	[D]Abnormal loss of weight (situation)
weight_loss	453341000000000	[D]Cachexia NOS (situation)
weight_loss	207558009	[D]Cachexia (situation)
weight_loss	207560006	[D]Cachexia NOS (situation)
weight_loss	23712001	Abnormal decrease in weight (finding)
weight_loss	238108007	Cachexia (finding)
weight_loss	271878009	Wasting disease (finding)
weight_loss	422868009	Unexplained weight loss (finding)
weight_loss	139091004	Weight loss (& abnormal) (finding)
weight_loss	158737005	[D]Cachexia (situation)
weight_loss	161832001	Weight decreasing (finding)
weight_loss	267024001	Abnormal weight loss (finding)
weight_loss	699205002	Involuntary weight loss (finding)
weight_loss	511461000000000	Unexplained weight loss (finding)
weight_loss	768581000000000	Unintentional weight loss
weight_loss	768571000000000	Unintentional weight loss (finding)
weight_loss	158271000	[D]Abnormal loss of weight (situation)
weight_loss	28928000	Cachexia (disorder)
weight_loss	162684007	On examination - cachexic (disorder)
