# Supplementary material for: Primary care referrals and abdominal imaging in people with new-onset diabetes and weight loss: cohort study using the English national sentinel network database
Source: BMJ Open. 2026 Aug 18;16(8):e119662. doi: 10.1136/bmjopen-2026-119662 (PMC13504520; doi:10.1136/bmjopen-2026-119662)
Supplement: Supplementary file 2 [file bmjopen-2026-119662.reviewer_comments.pdf]

PEER REVIEW HISTORY

BMJ Open publishes all reviews undertaken for accepted manuscripts. Reviewers are asked to complete a checklist review form and are provided with free text boxes to elaborate on their assessment. These free text comments are reproduced below.

ARTICLE DETAILS

Title (Provisional)

Primary care referrals and abdominal imaging in people with new-onset diabetes and weight loss: cohort study using the English national sentinel network database

Authors

Lemanska, Agnieszka; Claridge, Hugh; Price, Claire A; Abell, Jessica; Costello, Eithne; Evans-Lilley, Georgina; Pandol, Stephen; Richards, Poppy; Stolzenberg-Solomon, Rachael Z.; de Lusignan, Simon

---

VERSION 1 - REVIEW

**Reviewer**	**1**
**Name**	**Finelli, Carmine**
**Affiliation**	**3Center of Obesity and Eating Disorders, Stella Maris Mediterraneum Foundation, C/da S. Lucia, Chiaromonte, 80035 Potenza, Italy , Center of Obesity and Eating Disorders**
**Date**	**10-Apr-2026**
**COI**	**None**

Comments to Authors

This study showed opportunities to improve referral rates for people with NOD and weight loss. It suggested potential underutilisation of NICE referral guidelines and a possible gap in care and data quality. In June 2025, the NHS has launched an initiative to test ways to improve primary care referrals for abdominal imaging to assess for pancreatic cancer in highrisk patients.

Given that pancreatic cancer (PC) is typically diagnosed at an advanced stage but is often preceded by new-onset diabetes mellitus (NODM), providing a window for early detection, developed an interpretable ML framework centered on NODM enables practical risk stratification for early PC detection by multi-omics and provides a pathway of ML-based triage followed by biomarker confirmation for earlier detection and diagnosis [1]. NODM, identifiable in real time using active surveillance of electronic health records, is associated with a high 3-year incidence of pancreatic cancer with marked racial and ethnic differences [2].

Authors are kindly requested to emphasize the current concepts about these issues in the context of recent knowledge and the available literature. This articles should be quoted in the References list.

References

1. Machine learning-based clinical prediction model and multi-omics integration for assessing pancreatic cancer risk in new-onset diabetes. J Transl Med. 2026; 24 (1): 298. Published 2026 Jan 30. doi:10.1186/s12967-026-07767-1.

2. Risk of Pancreatic Cancer in Glycemically Defined New-Onset Diabetes: A Prospective Cohort Study. Gastroenterology. 2026; 170 (1): 106-117. doi:10.1053/j.gastro.2025.06.025.

**Reviewer**	**2**
**Name**	**Tkachenko, Victoria I**
**Affiliation**	**Bogomolets National Medical University, Internal and Family Medicine**
**Date**	**12-May-2026**
**COI**	**None**

The title is about the referrals and abdominal imaging in people with NOD, but actually the more important problem is discussed in the text of the article, it is about screening/early diagnosis of pancreatic cancer. The aim of the article has to be clearer. It is mentioned by authors "The aim of this study was to provide baseline data on referral and imaging rates in the target population". It is not understandable for reader what purpose of the referral is, what kind of referral and why the abdominal imaging is needed. The suggestions are to revise the title, aim and conclusion. They need to correlate to each other, to be clearer and more attractive for the reader, to indicate and to attract the attention to the problem of pancreatic cancer. And the aim (the research question) has to receive the answer in conclusion. For the reader it is also important to know what kind of abdominal imaging is needed according to NICE guidelines - US or CT, and how often it is needed to monitor HbA1c, BMI and other indexes, that you analysed, to understand the approaches in UK and how they are followed by GPs. The authors should add this information in discussion.

**Reviewer**	**3**
**Name**	**Blettner, Maria**
**Affiliation**	**Institute of Medical Biostatistics, Epidemiology and Informatics, University Medical Center, Johannes-Gutenberg University Mainz**
**Date**	**13-Jun-2026**

COI **None**

Major comment

This is an important and interesting dataset that could be used to investigate referral patterns and compliance with UK clinical recommendations regarding imaging for pancreatic cancer in high-risk groups.

The current analysis is largely descriptive. Additional subgroup analyses would be valuable to better understand factors associated with compliance to improve recommendations. For example:

Are there differences in referral rates between men and women, between ethnic groups, between regions?

As Index of Multiple Deprivation (IMD) data are available: do referral rates differ across IMD categories?

The age groups cover relatively wide age ranges. Are there further differences within older age groups, particularly among patients aged ≥60 years, for example 60–69, 70–79, and ≥80 years?

Such analyses may provide useful information for improving implementation of the recommendations.

Table 1:

I had some problems in understanding the numbers:

The overall dataset includes 181,055 individuals with diabetes, of whom 810 were diagnosed with pancreatic cancer. However, Table 1 reports 180,815 individuals included in the analysis. Please clarify this discrepancy.

Please check the confidence intervals reported for age. They appear unexpectedly narrow when compared with the median and interquartile range.

Please explain how individuals diagnosed with pancreatic cancer after the index date (n = 570) were identified and recorded. Was that during the first visit or during the complete follow-up period?

Methods

Page 5, line 31

Please explain why data collection started in January 2015, whereas implementation of the guideline began in June 2015. If the implementation only started in 2015, it may be useful to start the analysis somewhat later

Page 5, line 44

Restricting the analysis to practices that met strict data-quality criteria may introduce selection bias. This potential limitation should be discussed.

Page 6, line 5

• Were patients with cancers other than pancreatic cancer included in the study population? Please clarify.

Page 6, line 21

• The term “12-month window before and after” is unclear. Does this refer to a 24-month observation period (12 months before and 12 months after the index date), or another timeframe?

Results

Page 7, lines 21–28

• Please check and clarify the reported numbers, particularly in relation to the discrepancies noted in Table 1.

Discussion / Interpretation

Page 9 and Table 3

• It is unclear how the results presented in Table 3 contribute to the main analysis and support the study conclusions. Please clarify the role of these findings and explain how they inform the interpretation of referral patterns and guideline adherence.

VERSION 1 - AUTHOR RESPONSE

Reviewer: 1<br>Dr. Carmine Finelli, 3Center of Obesity and Eating Disorders, Stella Maris Mediterraneum Foundation, C/da S. Lucia, Chiaromonte, 80035 Potenza, Italy<br>Comments to the Author:	
This study showed opportunities to improve referral rates for people with NOD and weight loss. It suggested potential underutilisation of NICE referral guidelines and a possible gap in care and data quality. In June 2025, the NHS has launched an initiative to test ways to improve primary care referrals for abdominal imaging to assess for pancreatic cancer in high risk patients.	Thank you for providing the review.

Given that pancreatic cancer (PC) is typically diagnosed at an advanced stage but is often preceded by new-onset diabetes mellitus (NODM), providing a window for early detection, developed an interpretable ML framework centered on NODM enables practical risk stratification for early PC detection by multi-omics and provides a pathway of ML-based triage followed by biomarker confirmation for earlier detection and diagnosis [1]. NODM, identifiable in real time using active surveillance of electronic health records, is associated with a high 3-year incidence of pancreatic cancer with marked racial and ethnic differences [2].	This is a relent additional context – thank you for providing it.<br><br>We added the following text in the Discussion Section.<br><br>"Emerging approaches have combined NOD with interpretable machine learning and multi-omics data to improve pancreatic cancer risk stratification, suggesting that integration of routinely collected primary care data with these methods may offer opportunities for future data-driven approaches to enhance early detection.37"<br><br>"A recent study by Chari et al. (2026) confirmed the increased 3-year incidence of pancreatic cancer following NOD and identified important racial and ethnic differences in risk.38 These findings support the use of primary care data for further risk stratification based on demographic characteristics and targeted surveillance of groups at high risk."
Authors are kindly requested to emphasize the current concepts about these issues in the context of recent knowledge and the available literature. This articles should be quoted in the References list.<br><br>References<br>1. Machine learning-based clinical prediction model and multi-omics integration for assessing pancreatic cancer risk in new-onset diabetes. J Transl Med. 2026; 24 (1): 298. Published 2026 Jan 30. doi:10.1186/s12967-026-07767-1.<br>2. Risk of Pancreatic Cancer in Glycemically Defined New-Onset Diabetes: A Prospective Cohort Study. Gastroenterology. 2026; 170 (1): 106-117. doi:10.1053/j.gastro.2025.06.025.	Done<br><br>The suggested references were added to the reference list.

<table>
<tr><th colspan="2">Reviewer: 2
Dr. Victoria I Tkachenko, Bogomolets National Medical University
Comments to the Author:</th></tr>
<tr><td>The title is about the referrals and abdominal imaging in people with NOD, but actually the more important problem is discussed in the text of the article, it is about screening/early diagnosis of pancreatic cancer. The aim of the article has to be clearer.</td><td>Thank you for providing the review.

We appreciate that the broader clinical context of the study is ultimately to investigate approaches for improving early detection of pancreatic cancer. However, the primary aim of this study was not to evaluate screening or diagnostic strategies for pancreatic cancer. It was to report referral rates and data quality:

“UK clinical guidelines recommend abdominal imaging to rule out pancreatic cancer in this high-risk group. In June 2025, National Health Service (NHS) England launched a case-finding pilot to test approaches to improve primary care referrals for imaging in these patients and improve the quality of body weight and diabetes-related data recorded in electronic healthcare records (EHRs). This study reports referral and imaging rates among the eligible group and the availability of body weight and glycated haemoglobin (HbA1c) measurements in people with diabetes, providing baseline data prior to implementation of the NHS pilot.”

Therefore, in a specific impact context, this study was not to improve early detection or screening, but to support the implementation and evaluation the NHS pancreatic cancer case finding pilot.</td></tr>
<tr><td>It is mentioned by authors "The aim of this study was to provide baseline data on referral and imaging rates in the target population". It is not understandable for reader what purpose of the referral is, what kind of referral and why the abdominal imaging is needed.</td><td>Done. Thank you. We clarified the aim statement at the and of the Introduction Section and it now reads:

“The aim of this study was to provide baseline data on referral rates for suspected pancreatic cancer and abdominal imaging, computed</td></tr>
</table>

	tomography (CT) and ultrasound scans (USS) in the target population.”
The suggestions are to revise the title, aim and conclusion. They need to correlate to each other, to be clearer and more attractive for the reader, to indicate and to attract the attention to the problem of pancreatic cancer. And the aim (the research question) has to receive the answer in conclusion.	In the first instance, the Discussion Section addresses the research question by evaluating how closely general practices followed the NICE NG12 pancreatic cancer referral guideline for individuals with NOD and weight loss.<br><br>It also explains that the study provides baseline evidence on clinical practice and data recording to support the NHS England initiative aimed at improving uptake of the referral guideline. Furthermore, it demonstrates how routinely collected data can be used to evaluate compliance with, and the impact of, national clinical guidelines. Establishing this baseline highlighted gaps in data quality and clinical practice, providing a foundation for the NHS to target interventions that support earlier pancreatic cancer diagnosis.<br><br>As highlighted by the reviewer, the Conclusions section states that the study identified opportunities to improve referral rates for people with NOD and unexplained weight loss. It suggests potential underutilisation of the NICE referral guideline and identifies possible gaps in care and data quality. We believe these points directly address the stated aims and objectives of the study.
For the reader it is also important to know what kind of abdominal imaging is needed according to NICE guidelines - US or CT,	Done<br><br>Box 1 in the manuscript states as follows:<br><br>“Consider an urgent, direct access computed tomography (CT) scan or an urgent ultrasound scan if CT not available, to assess for pancreatic cancer in people aged 60 and over with weight loss and any of the following:<br>• diarrhoea<br>• back pain<br>• abdominal pain

	• nausea<br>• vomiting<br>• constipation<br>• new-onset diabetes (added in 2015)”
and how often it is needed to monitor HbA1c, BMI and other indexes, that you analysed, to understand the approaches in UK and how they are followed by GPs. The authors should add this information in discussion.	Done<br><br>Thank you for this request. We agree, this additional context helps readers interpret the data on completeness of the routinely recorded measurements reported in our study.<br><br>We have added the following text to the Discussion clarifying that. The manuscript now states:<br><br>“In UK primary care, body weight, and HbA1c are routinely collected clinical measures. For people with diabetes, these assessments are recommended as part of annual diabetes reviews and should be recorded at least once a year, although more frequent monitoring may be undertaken according to individual clinical need.”
Reviewer: 3<br>Dr. Maria Blettner, Institute of Medical Biostatistics, Epidemiology and Informatics, University Medical Center, Johannes-Gutenberg University Mainz<br>Comments to the Author:	
Major comment<br>This is an important and interesting dataset that could be used to investigate referral patterns and compliance with UK clinical recommendations regarding imaging for pancreatic cancer in high-risk groups. The current analysis is largely descriptive.	Thank you for providing a thorough review and a detailed set of constructive suggestions to improve the clarity and quality of the manuscript.
Additional subgroup analyses would be valuable to better understand factors associated with compliance to improve recommendations. For example:<br>Are there differences in referral rates between men and women, between ethnic groups, between regions?<br>As Index of Multiple Deprivation (IMD) data are available: do referral rates differ across IMD categories? The age groups	Thank you for making this important point on further cohort stratification. We agree that further subgroup analyses, for example by sex, ethnicity, geographic region, socioeconomic deprivation and narrower age categories, could provide additional insights into variation in clinical practice and healthcare inequalities.

cover relatively wide age ranges. Are there further differences within older age groups, particularly among patients aged ≥60 years, for example 60–69, 70–79, and ≥80 years? Such analyses may provide useful information for improving implementation of the recommendations.	We added the following text in the Discussion Section on Implications and future research:<br><br>A recent study by Chari et al. (2026) confirmed the increased 3-year incidence of pancreatic cancer following NOD and identified important racial and ethnic differences in risk.[38] These findings support the use of primary care data for further risk stratification based on demographic characteristics and targeted surveillance of groups at high risk. Future analyses should examine variation in referral and abdominal imaging rates by sex, ethnicity, geographic region and IMD to identify demographic factors associated with guideline adherence and opportunities to improve equitable implementation.<br><br>For this study specifically, these analyses were beyond the scope and were not specified in the original study protocol or ethics application, which focused on providing national estimates of referral and imaging rates to support the implementation of the NHS England programme.
Table 1:<br>I had some problems in understanding the numbers:<br>The overall dataset includes 181,055 individuals with diabetes, of whom 810 were diagnosed with pancreatic cancer. However, Table 1 reports 180,815 individuals included in the analysis. Please clarify this discrepancy.	Done<br><br>We have revised rows 3 and 4 of Table 1. Please see the tracked changes version of the manuscript.<br><br>We first reported the full cohort of 181,055 individuals diagnosed with diabetes. This was because the date of diabetes diagnosis was defined as the study index date. However, the final study cohort excluded 240 individuals diagnosed with pancreatic cancer before their diabetes diagnosis. Therefore, the final analysis cohort comprised 180,815 individuals and 570 pancreatic cancer diagnoses after the index data (810 pancreatic cancer diagnosis in total).

	This has been clarified in the text, please see the sentence highlighted in yellow in the tracked version of the manuscript (Methods, Study cohort Subsection) which states:<br><br>"Individuals with unspecified diabetes type, gestational diabetes, and pancreatic cancer diagnosis before diabetes were excluded."
Please check the confidence intervals reported for age. They appear unexpectedly narrow when compared with the median and interquartile range.	Checked<br><br>We have checked the calculations and the analytical script, and the reported 95% CIs are correct. Although the interquartile ranges indicate substantial variability in individual ages, the confidence intervals relate to the precision of the estimated mean and given the large sample size of over 180,000 participants overall and around 90,000 in each age subgroup, the standard errors of the mean were very small, resulting in narrow 95% confidence intervals. We reported both the mean (95% CI) and the median (IQR) to provide this complementary information and enable comparisons with other studies which report just one estimate.
Please explain how individuals diagnosed with pancreatic cancer after the index date (n = 570) were identified and recorded. Was that during the first visit or during the complete follow-up period?	Done<br><br>We added the following text in the revised manuscript.<br><br>"The dates of diabetes and pancreatic cancer diagnoses were defined as the first recorded entry of the relevant clinical code. These dates were used to determine eligibility for the study cohort, and individuals with a recorded pancreatic cancer diagnosis before their diabetes diagnosis were excluded."
Methods<br>Page 5, line 31	Done

Please explain why data collection started in January 2015, whereas implementation of the guideline began in June 2015. If the implementation only started in 2015, it may be useful to start the analysis somewhat later	The revised sentence in the manuscript now states:<br><br>"The study period began in 2015 because of the implementation of the NG12 guidelines in June 2015, recognising that adoption of new guidelines is gradual rather than immediate."<br><br>The choice of January 2015 as the study start date was arbitrary and pragmatic and provided a clear calendar-based cut-off close to the publication of the NICE NG12 guideline in June 2015. This is pragmatic in the context that we recognise that implementation of new clinical guidelines is not a clear-cut one-off event. The NG12 recommendations were developed over an extended period and were already being discussed within clinical and professional communities before their formal publication.<br><br>Our clinical collaborators indicated that restricting the study to patients diagnosed only after June 2015 or after a later implementation period would limit the data for year 2015 and would not fully reflect the adoption of the guideline, it would also reduce the available sample size. We have clarified this rationale in the Methods section.
Page 5, line 44<br>Restricting the analysis to practices that met strict data-quality criteria may introduce selection bias. This potential limitation should be discussed.	Done<br><br>A sentence was added in the Limitations Section.<br>"Inclusion was restricted to practices meeting predefined data quality standards, which may have introduced selection bias if clinical practice in these practices differed systematically from those not meeting the data quality standards."
Page 6, line 5<br>• Were patients with cancers other than pancreatic cancer included in the study population? Please clarify.	We added the following sentence:<br><br>"Information on cancers other than pancreatic cancer was not extracted, and

	individuals with other cancer diagnoses were eligible for inclusion in the study.”
Page 6, line 21<br>• The term “12-month window before and after” is unclear. Does this refer to a 24-month observation period (12 months before and 12 months after the index date), or another timeframe?	This has been revised and it now states:<br><br>“A 24-month study window, comprising the 12 months before and the 12 months after the index date, was applied to ...”
Results<br>Page 7, lines 21–28<br>• Please check and clarify the reported numbers, particularly in relation to the discrepancies noted in Table 1.	We have highlighted in yellow the text which describes the derivation of the final study cohort. Also as requested, we clarified in Table 1 that individuals diagnosed with pancreatic cancer before their diabetes diagnosis (the study index date) were excluded.<br><br>The manuscript states:<br><br>" There were 181,055 adults diagnosed with diabetes between January 2015 and May 2021 in 781 general practices. Of these, 810 (0.4%) were diagnosed with pancreatic cancer, with 127 (0.1%) in the < 60 age group and 683 (0.7%) in the ≥ 60 age group. ... Within the 810 cancer diagnoses, 240 occurred before the diagnosis of diabetes and these individuals were excluded from further analysis.<br><br>The final study cohort consisted of 180,815 people, with 88,605 (49.0%) in the < 60 age group and 92,210 (51.0%) in the ≥ 60 age group (Table 1)."
Discussion / Interpretation<br>Page 9 and Table 3<br>• It is unclear how the results presented in Table 3 contribute to the main analysis and support the study conclusions. Please clarify the role of these findings and explain how they inform the interpretation of referral patterns and guideline adherence.	Table 3 is linked to the secondary aim of the study<br><br>The abstract states:<br><br>“This study reports referral and imaging rates among the eligible group and the availability of body weight and glycated haemoglobin (HbA1c) measurements in people with diabetes, providing baseline data prior to implementation of the NHS pilot.”

	The last paragraph of the Introduction Section (highlighted) provides a clarification on the role of these analyses and how they inform the interpretation of the study contributing to its overarching aim to support the roll out and the evaluation of the new NHS pancreatic cancer case-fining pilot. (one of the aims of the NHS pilot is to improve the quality of the NHS data)<br><br>"Referral rates and abdominal imaging were assessed among individuals with NOD and unexplained weight loss, along with the completeness of body weight and glycated haemoglobin (HbA1c) data. We assess the latter because one of the objectives of the NHS trial is to improve the recording of body weight and HbA1c in EHRs. Regular weight and HbA1c recordings are important, because they can be used to determine eligibility for a referral. Following completion of the NHS trial, these analyses will be repeated to assess changes in referral and imaging rates, pancreatic cancer diagnoses, and data quality, complementing the NHS trial findings with real-world evidence from practice."

VERSION 2 - REVIEW

**Reviewer**	**2**
**Name**	**Tkachenko, Victoria I**
**Affiliation**	**Bogomolets National Medical University, Internal and Family Medicine**
**Date**	**31-Jul-2026**
**COI**	

The article is strong and represents the results at national level, the results are very importat for improvement of health system and pancreatic cancer prevention. All reviewers' suggestions were taken into account, so my recommedation is to accept.

Reviewer 3

Name Blettner, Maria

Affiliation Institute of Medical Biostatistics, Epidemiology and Informatics, University Medical Center, Johannes-Gutenberg University Mainz

Date 24-Jul-2026

COI

All my questions have been answered and clarified.
